# *Psoralea corylifolia* L.: a comprehensive review of its botany, traditional uses, phytochemistry, pharmacology, toxicology, quality control and pharmacokinetics

**DOI:** 10.1186/s13020-022-00704-6

**Published:** 2023-01-10

**Authors:** Lele Chen, Shuguang Chen, Peng Sun, Xinyue Liu, Zhaoshuang Zhan, Jiafeng Wang

**Affiliations:** College of Chinese Medicine, Shandong University of Traditional Chinese Medicine, No.4655 Daxue Road, Jinan, 250355 China

**Keywords:** *Psoralea corylifolia* L., Traditional uses, Phytochemistry, Pharmacology, Toxicology, Quality control

## Abstract

*Psoralea corylifolia* L. (PCL), referred to as “Bu-gu-zhi” in Chinese, has great medicinal values since ancient times. PCL is the dried ripe fruit of *Psoralea corylifolia* L., which has been widely used in traditional Chinese medicine (TCM) for the treatment of kidney-yang deficiency, enuresis and urinary frequency, chills and pain of the waist and knees, dawn diarrhea and vitiligo. In this paper, a systematic of the botany, traditional uses, phytochemistry, pharmacology, toxicology, quality control and pharmacokinetics of PCL was presented, along with future research directions. According to the results, PCL contains approximately 163 chemical components, including coumarins, flavonoids, monoterpene phenols, benzofurans, glycosides, lipids, fatty acids, and volatile oils. PCL and its active ingredients have a variety of pharmacological activities, such as anti-inflammatory, antibacterial, antiviral, antioxidant, antitumor, antiosteoporosis, cardioprotective, neuroprotective, and immunomodulatory. Further study of quality control standards and potential mechanisms of PCL is also needed. In addition, more toxicological studies will also contribute to the progress of clinical trials.

## Introduction

*Psoralea corylifolia* L. (PCL) is one of the most commonly used traditional Chinese medicines (TCMs) in China. Its Chinese name, “Buguzhi”, comes from the dried ripe fruit of *Psoralea corylifolia* L., a significant member of the family Leguminosae of genus *Psoralea* Linn. The fruit is harvested in the autumn, dried in the sun, and served raw or stir-fried with salt. PCL was first described in “Lei’s Treatise on Preparing Drugs” (《雷公炮炙论》Northern and Southern Dynasties, A.D. 420–581). It is warm in nature, pungent and bitter to the taste, and acts on the spleen and kidney meridians. It reinforces kidney and strengthens yang, helps inspiration and relieves asthma, warms spleen and stops diarrhea, secures essence and reduces urination, and can be applied externally to get rid of wind and freckles [1]. PCL has a wide geographic spread throughout China, particularly in the provinces of Yunnan, Sichuan, Guangxi, Henan, Anhui, Shanxi, and Guizhou. In these areas, it is either cultivated or wild, and it primarily grows on mountain slopes, beside streams, and by farms [2].

Numerous studies on PCL have been carried out recently by academics both domestically and internationally. PCL contains a variety of chemical elements, including coumarins, flavonoids, monoterpene phenols, benzofurans, glycosides, lipids, fatty acids, and volatile oils, according to phytochemical research. Modern pharmacological research has revealed that PCL has anti-inflammatory, antibacterial, antiviral, antioxidant, anti-tumor, photosensitizing and estrogen-like properties. Additionally, it has effect on the cardiovascular, nervous, immune, locomotor, and reproductive system, and has a broad application prospect. PCL can cause toxic reactions in the liver, kidney, skin and reproduction when administered long-term or excessively. Despite PCL’s long-term efficacy as an ethnomedicine, a comprehensive assessment of its safety, quality control and pharmacokinetics has not been conducted.

In this review, we present a comprehensive summary and analysis of the botany, traditional uses, phytochemistry, pharmacology, toxicology, quality control and pharmacokinetics of fresh PCL, as well as discuss the shortcomings of the existing research and make suggestions for potential future research directions. We hope to provide valuable references for further in-depth research, development and application of PCL.

## Botany

The morphological traits of PCL original plants are as follows: PCL is an erect annual herb that grows to a height of 60–150 cm, with a stiff branch covered with conspicuous white glandular spots and sparse white tomentose. Unifoliate, broadly ovate leaves measure approximately 4.5–9 cm long and 3–6 cm wide. There are obtuse or acute tips at the apex, a round or cordate base, a thick and irregularly serrated margin, and a tough texture with obvious black glandular dots on both sides and sparsely hairy or subglabrous cover. It has an axillary, dense raceme or tiny capitate inflorescence with 10–30 blooms. The involucre peduncle measures 3–7 cm in length and has glandular tips and white pubescence. 4–6 mm long, with a white pilose and glandular calyx, the bract is membranous, lanceolate, with tomentose and glandular dots. The petal has a noticeable stalk, and the corolla is yellow or blue. 10 stamens are separated distally. The pod has an irregularly reticulate surface and is black and indehiscent. The pericarp is difficult to separate from the seeds. From July to October, flowers and fruits are produced [2].

**Fig. 1:**
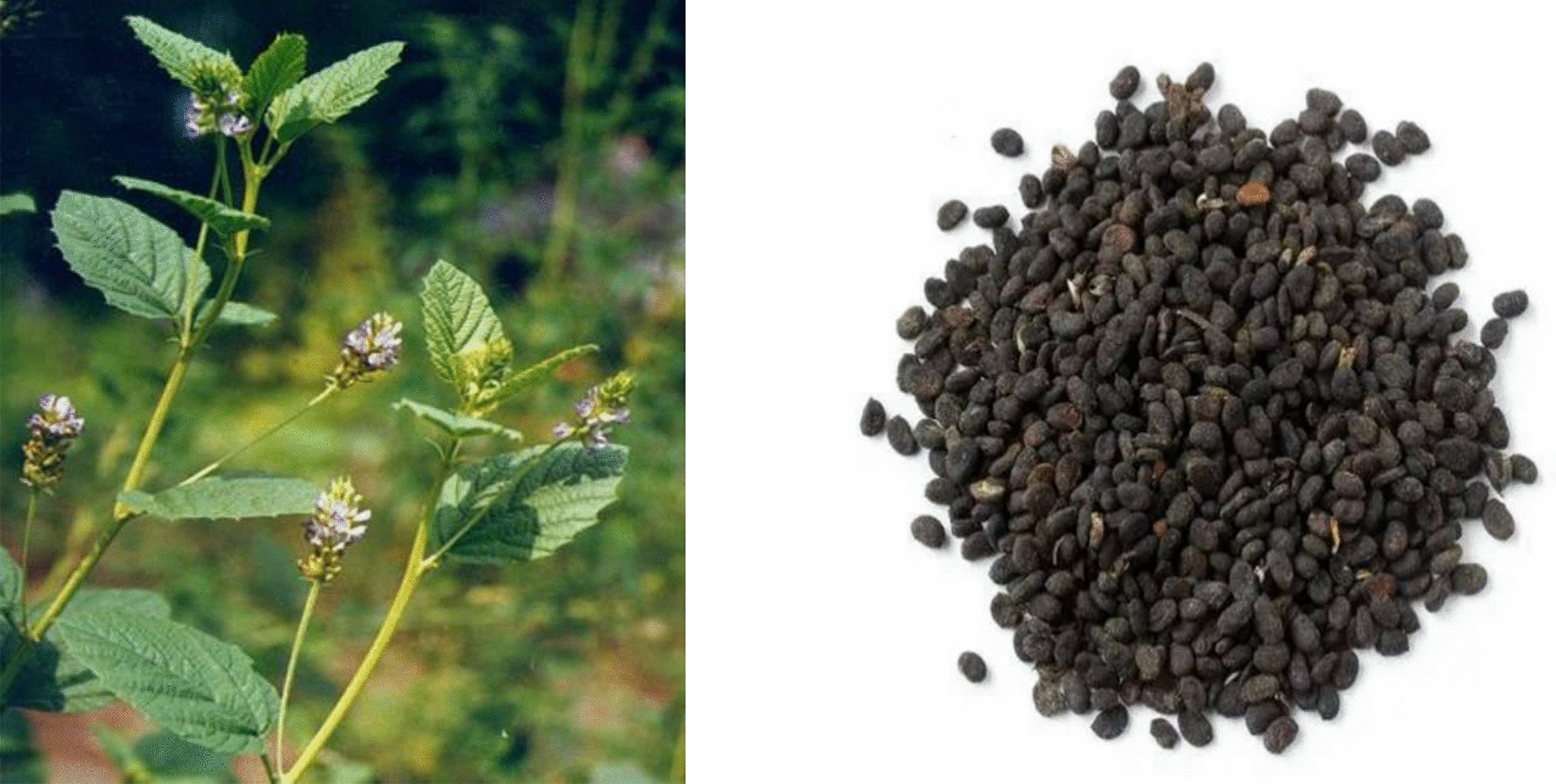
Original plants and commercial herbal pieces of PCL

The morphological characteristics of PCL commercial herbal pieces are reniform, slightly flattened, 3–5 mm long, 2–4 mm wide, and 1.5 mm thick; the surface is black, black-brown or grey-brown, with fine reticulate wrinkles, and the texture is hard. The top is round and obtuse, with a small protuberance and fruit stalks on the concave side. The pericarp is thin and difficult to separate from the seed; there is one seed with two greasy, yellow-white cotyledons, fragrant, pungent and slightly bitter [1]. The original plant and commercial herbal of PCL are shown in Fig. 1.

**Fig. 2:**
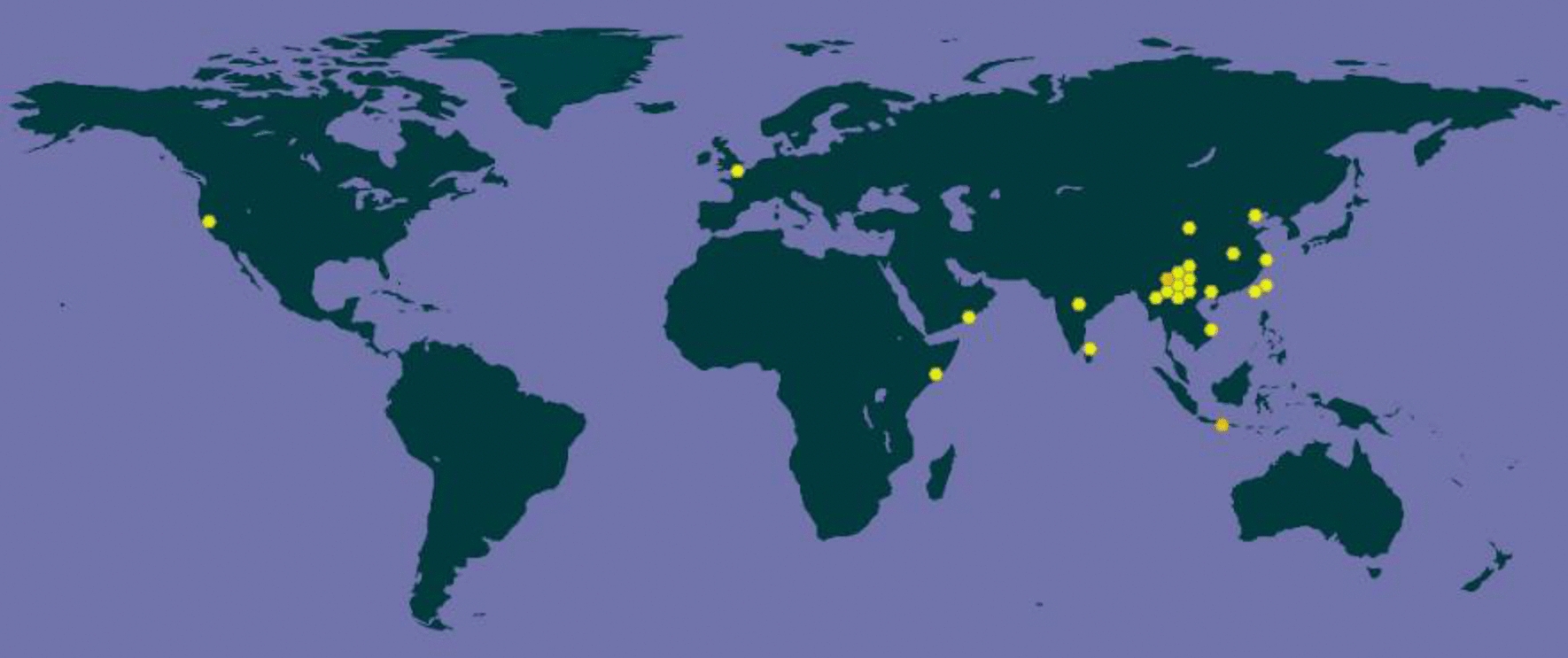
General geographical distribution of PCL in the world

The Psoralea genus has about 120 species, most of which are found in southern Africa, North and South America, and Australia. There are also a few in Asia and temperate Europe. China has one species, which is found in the provinces of Yunnan, Sichuan, Guangxi, Henan, Anhui, Shaanxi, and Guizhou. It likes a warm, humid, sunny environments and frequently grows along streams, in fields, and on mountain slopes [2]. We obtained the geographical distribution of PCL in the world from the GBIF online database (www.gbif.org, shown in Fig. 2).

## Traditional uses

The first mention of PCL can be found in “Lei’s Treatise on Preparing Drugs” (《雷公炮炙论》A.D. 420–581). The “Theory of Property” (《药性论》A.D. 618–907) which discussed PCL’s effectiveness, stated that it was mostly used for lumbar pain of males, cold knees and dampness. It was also used to relieve frequent urination and suppress coldness in the abdomen. According to “Herbal Medicines for Kaibao” (《开宝本草》A.D. 973–974), PCL was used for the treatment of five strains and seven injuries, wind deficiency and coldness, bone marrow injury and defeat, cold sperm flow from the kidney and abortion of females. “Rihuazi Materia Medica” (《日华子本草》A.D. 968–975) said that PCL could boost libido, relieve cold strain, and brighten the ears and eyes. PCL was also mentioned in other well-known traditional Chinese medicine publications, including the “Materia medica product Hui Jingyao” (《本草品汇精要》A.D. 1505), “Compendium of Materia Medica” (《本草纲目》A.D. 1552–1578), “Yuqiu Medicine Solution” (《玉楸药解》 A.D. 1754), and Chinese Pharmacopoeia (2020 edition), among others. It is important to note that PCL should not be taken by people who has a fire excess and a Yin deficiency.

These days, PCL is a widely used traditional Chinese medicine to treat kidney and spleen deficiencies. Different dose forms, including pills, decoctions, granules, tinctures, injections, and others, have been developed to be better administered in clinics. Some Chinese prescriptions mainly containing PCL are presented in Table 1. Among them, Bushen Yangxue Wan can be used to support the liver and kidney, as well as to improve blood and essence. Astringing the intestines to stop diarrhea, warming yang to activate qi flow, and treating chronic, ulcerative, and allergic colitis are all possible uses for Bupi Yichang Wan. Bufei Huoxue Jiaonang can be utilized to nourish the kidney and lung while stimulating qi and blood circulation. Treatment for vitiligo, flat warts, alopecia areata, and other skin conditions always involves the usage of Buguzhi Ding.

**Table 1:** Common traditional Chinese medicine prescriptions containing PCL

Preparation name	Type	Main compositions	Traditional and clinical uses	References
Bubai granules	Granules	Psoraleae Fructus, Semen Dolichoris Album, Epimedii Folium, Salviae Miltiorrhizae, Radix Bupleuri, Semen Sojae Atricolor, Semen Phaseoli, Sophorae Flavescentis Radix	Treating chronic leukopenia belongs to the spleen and kidney insufficiency	[1]
Bushen Yangxue Wan	Pills	Radix Polygoni Multiflori, Angelicae Sinensis Radix, Semen Sojae Atricolor, Radix Achyranthis Bidentatae, Poria, Cuscutae Semen, Psoraleae Fructus, Fructus Lycii	Treating bodily weakness, deficiency of qi and blood, seminal emission, premature graying hair	[1]
Bushen Yinao Wan	Pills	Cervi Cornu Pantotrichum, Red Ginseng, Poria, Dioscoreae Rhizoma, Rehmanniae Radix Praeparata, Angelicae Sinensis Radix, Chuanxiong Rhizoma, Psoraleae Fructus, Radix Achyranthis Bidentatae, Fructus Lycii, Scrophulariae Radix, Radix Ophiopogonis, Schisandrae Chinensis Fructus, Ziziphi Spinosae Semen, Polygalae Radix, Cinnabaris	Treating palpitations, short breath, forgetfulness, seminal emission, night sweating, lassitude in loin and legs, deafness and tinnitus	[1]
Bupi Yichang Wan	Pills	Outer layer: Astmgali Radix, Codonopsis Radix, Amomi Fructus, Paeoniae Radix Alba, Angelicae Sinensis Radix, Macrocephalae Rhizoma, Cinnamomi Cortex Inner layers: Corydalis Rhizoma, Litchi Semen, Common Ginger, Glycyrrhizae Radix Et Rhizoma Praeparata Cum Melle, Saposhnikoviae Radix, Aucklandiae Radix, Psoraleae Fructus, Halloysitum Rubrum	Treating diarrhea caused by spleen deficiency and Qi stagnation; Chronic colitis, ulcerative colitis and allergic colitis	[1]
Bufei Huoxue Jiaonang	Capsules	Astmgali Radix, Paeoniae Radix Rubra, Psoraleae Fructus	Treating pulmonary heart disease (remission) belongs to the syndrome of Qi deficiency and blood stasis	[1]
Buguzhi Wan	Pills	Psoraleae Fructus, Bitter Apricot Seed, Persicae Semen	Treating the lumbago is unbearable	[7]
Buguzhi Ding	Tinctures	Psoraleae Fructus, 75% alcohol	Treating vitiligo, verruca plana, alopecia areata, neurodermatitis and pruritus	[7]
Buguzhi Tang	Decoctions	Psoraleae Fructus, Aconiti Lateralis Radix Praeparata, Ginseng Radix Et Rhizoma, Cistanches Herba, Schisandrae Chinensis Fructus	Pain and weakness of bone	[7]
Buguzhi San	Powders	Psoraleae Fructus, Fennel, Semen Trigonellae, Arecae Semen, Citri Reticulatae Pericarpium Viride	Treating urine incontinence, abdominal colic	[7]
Buguzhi Jian	Decoctions	Psoraleae Fructus, Benzoinum, Juglandis Semen	Treating female morbid leucorrhea and feebleness	[7]
Buguzhi Zhusheye	Injections	Psoraleae Fructus	Treating vitiligo, psoriasis	[8]

According to modern pharmacological studies, in patients with leukopenia following tumor chemotherapy and radiation therapy, Bubai granules efficiently raised platelet count and neutrophil absolute value levels and markedly reduced clinical symptoms [3]. Buguzhi Tang could effectively treat femoral head necrosis, reduce blood viscosity, improve hemorheology, increase bone density, and improve hip joint function [4]. Furthermore, Buguzhi Tang could considerably enhance cognitive performance in vascular dementia patients who lack kidney-essence [5]. When coupled with Fructus Psoraleae, Halloysitum Rubrum and Sanchi could dramatically speed up nasal hemostasis and lessen bleeding [6]. However, multiplex interactions and molecular mechanisms between PCL and compatible medicinal plants remain unclear and may be studied in the future.

To ensure safety, enhance efficacy and reduce toxicity, PCL is usually processed before clinical use. The earliest method of PCL processing was recorded in the “Lei’s Treatise on Preparing Drugs” (《雷公炮炙论》A.D. 420–581), in which the processing method of PCL was soaked in wine and water respectively, and dried in the sun. According to “Rihuazi Materia Medica” (《日华子本草》A.D. 968–975) PCL in therapeutic settings needed to be lightly stir-fried. Currently, PCL’s main processing methods are taking the original herb, removing impurities and salt-processing it [1]. Modern studies have demonstrated that the composition and efficacy of PCL change during processing, which may be the mechanism for reducing toxicity and enhancing efficacy of PCL processing. However, it needs further investigation.

## Phytochemistry

**Fig. 3:**
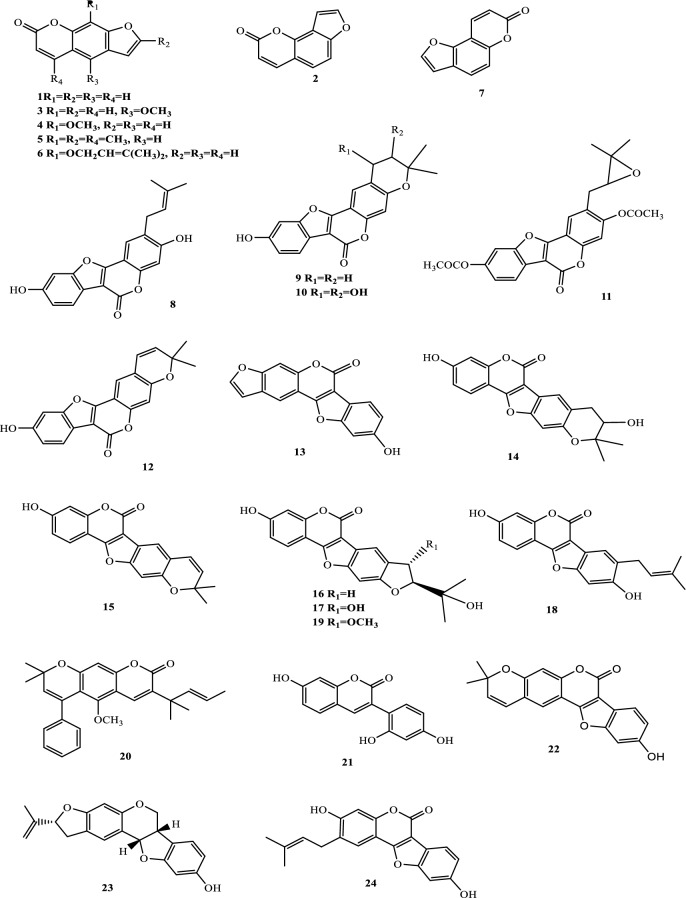
Chemical structures of coumarins isolated from PCL

**Fig. 4:**
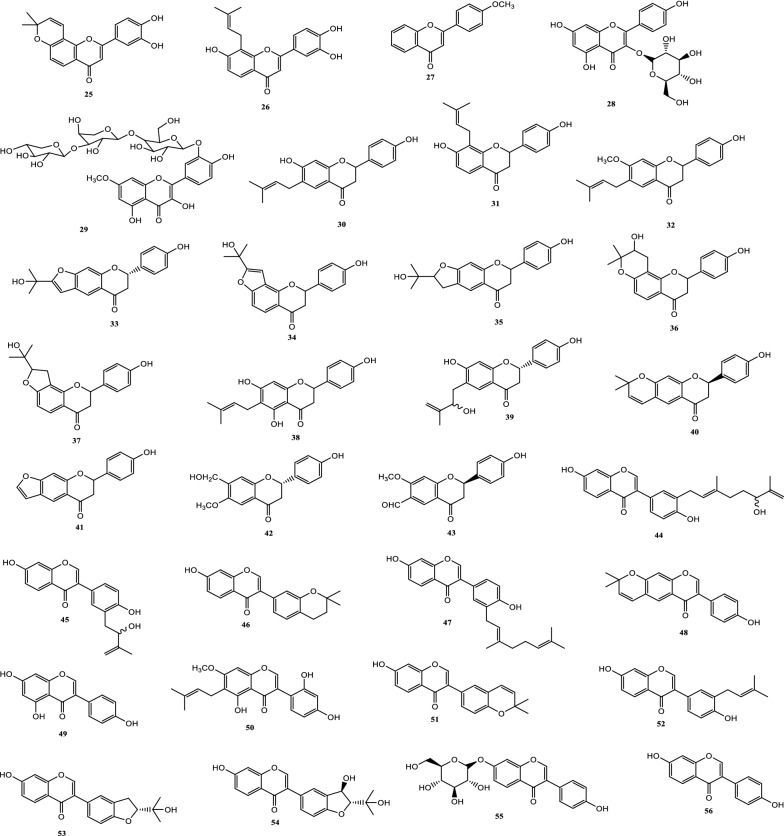

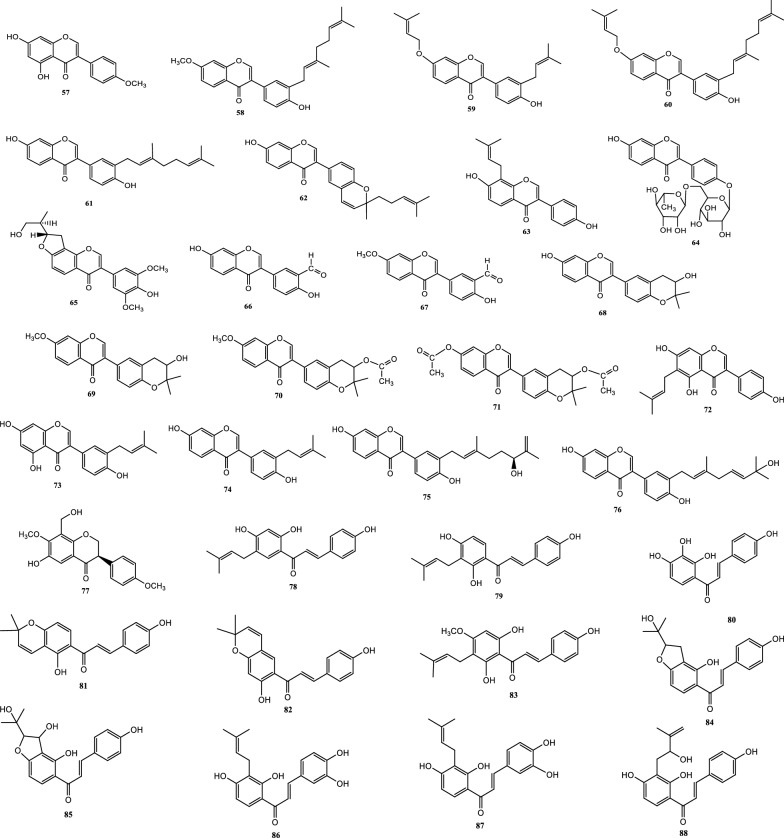

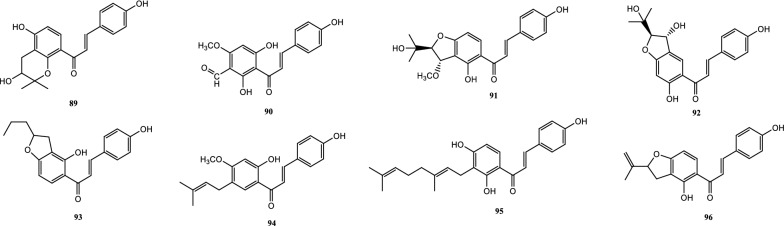
Chemical structures of flavonoids isolated from PCL

**Fig. 5:**
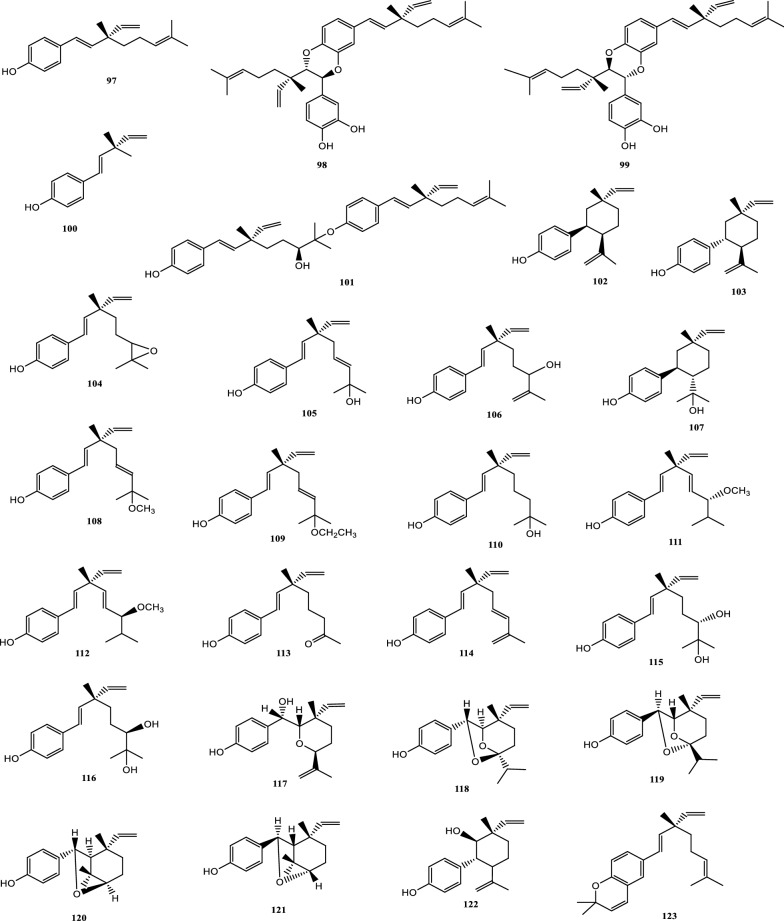

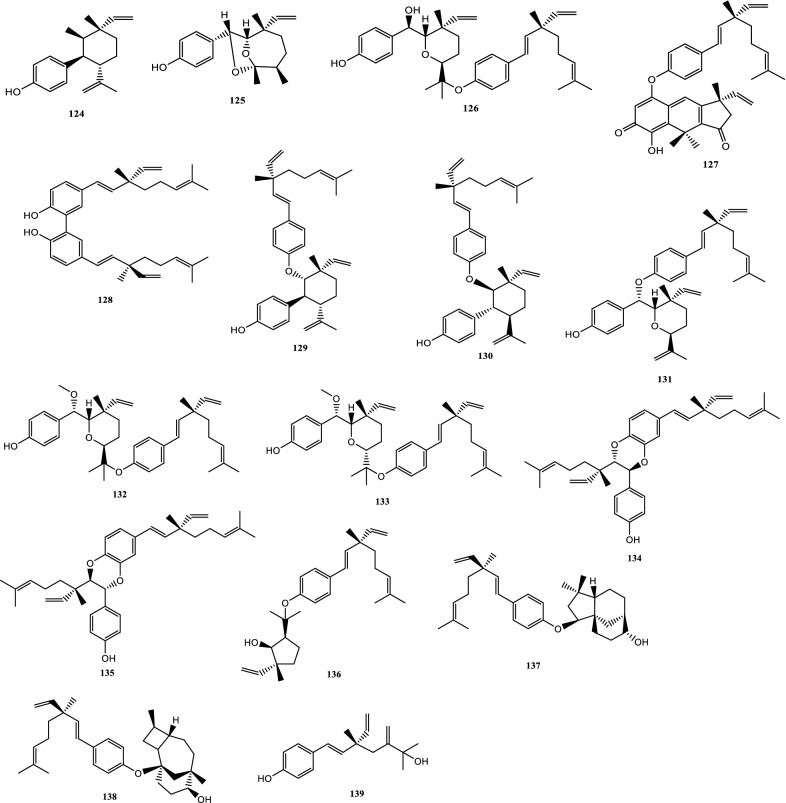
Chemical structures of monoterpene phenols isolated from PCL

**Fig. 6:**
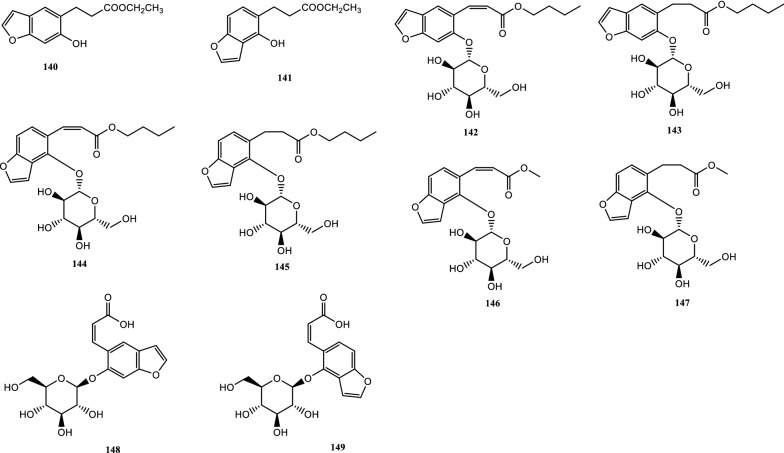
Chemical structures of benzofurans isolated from PCL

**Fig. 7:**
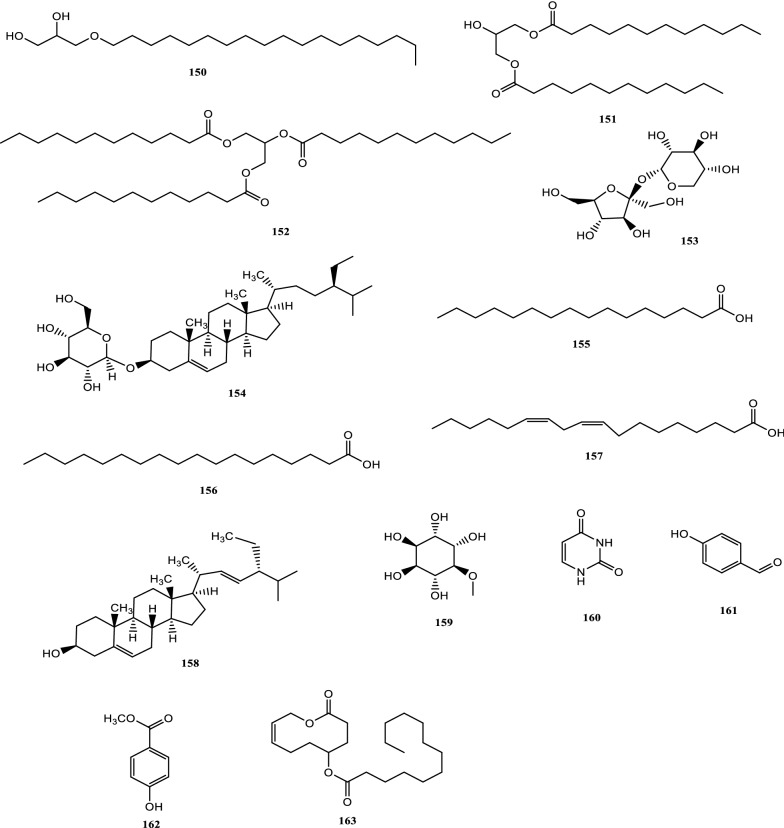
Chemical structures of other compounds isolated from PCL

Drugs’ pharmacological actions are carried out by their chemical components. Over 163 chemicals have so far been identified from fresh PCL. A number of phytochemical studies have revealed that PCL primarily contains coumarins (1–24), flavonoids (25–96), monoterpene phenols (97–139), benzofurans (140–149) and other compounds (150–163). Among them, coumarins, flavonoids and monoterpene phenols are considered to be the PCL’s primary bioactive components. These compounds are crucial for a variety of pharmacological actions and therapeutic outcomes. In this article, we summarized all compounds identified in PCL and showed in Table 2, along with their corresponding structures in Figs. 3, 4, 5, 6 and 7.

**Table 2:** Compounds isolated from PCL

Class	NO	Chemical component	Chemical formula	References
Coumarins	1	psoralen	C_11_H_6_O_3_	[9]
	2	isopsoralen	C_11_H_6_O_3_	[10]
	3	5-methoxy psoralen	C_12_H_8_O_4_	[10]
	4	8-methoxy psoralen	C_12_H_8_O_4_	[10]
	5	4,5ʹ,8-trioxsalen	C_14_H_12_O_3_	[11]
	6	imperatorin	C_16_H_14_O_4_	[11]
	7	bakuchicin	C_11_H_6_O_3_	[12]
	8	psoralidin	C_20_H_16_O_5_	[13]
	9	isopsoralidin	C_20_H_16_O_5_	[13]
	10	corylidin	C_20_H_16_O_7_	[14]
	11	psoralidin-2ʹ,3ʹ-oxide	C_24_H_20_O_8_	[15]
	12	4ʺ, 5ʺ-dehydroisopsoralidin	C_20_H_14_O_5_	[16]
	13	neopsoralen	C_17_H_8_O_5_	[17]
	14	bvacoumestan A	C_20_H_16_O_6_	[18]
	15	sophoracoumestan A	C_20_H_14_O_5_	[18]
	16	bvacoumestan B	C_20_H_16_O_6_	[18]
	17	bavacoumestan C	C_20_H_16_O_7_	[19]
	18	bavacoumestan D	C_20_H_16_O_5_	[19]
	19	3ʺ-methoxy-bavacoumestan C	C_21_H_18_O_7_	[20]
	20	pyranocoumarin	C_27_H_28_O_4_	[21]
	21	7,2ʹ,4ʹ-trihydroxy-3-aryl-coumarin	C_15_H_10_O_5_	[22]
	22	psoracoumestan	C_20_H_14_O_5_	[22]
	23	seputhecarpan A	C_20_H_18_O_4_	[23]
	24	psoralidinl	C_20_H_16_O_5_	[24]
Flavonoids	25	corylifol D	C_20_H_16_O_5_	[25]
	26	corylifol C	C_20_H_18_O_5_	[26]
	27	4ʹ-methoxyflavone	C_16_H_12_O_3_	[27]
	28	astragalin	C_21_H_20_O_11_	[10]
	29	3,5,3ʹ,4ʹ-tetrahydroxy-7-methoxyflavone-3ʹ-o-α-l-xylopyranosyl (1 → 3)-o-α-l-arabinopyranosyl (1 → 4)-o-β-d-galactopyranoside	C_32_H_38_O_20_	[28]
	30	bavachin	C_20_H_20_O_4_	[29]
	31	isobavachin	C_20_H_20_O_4_	[29]
	32	bavachinin A	C_21_H_22_O_4_	[29]
	33	6,7-furanbavachinone B	C_20_H_18_O_5_	[20]
	34	bavachinone B	C_20_H_18_O_5_	[30]
	35	bavachinone A	C_20_H_20_O_5_	[30]
	36	brosimacutin D	C_20_H_20_O_5_	[24]
	37	brosimacutin E	C_20_H_20_O_5_	[24]
	38	6-prenylnaringenin	C_20_H_20_O_5_	[31]
	39	bakuisoflavone	C_20_H_18_O_5_	[32]
	40	7, 8-Dihydro-8- (4-hydroxyphenyl) -2, 2-dimethyl-2H, 6H-[1, 2-b:5, 4-b]dipyran-6’-one)	C_20_H_18_O_4_	[33]
	41	furano (2ʺ,3ʺ,7,6)-4ʹ-hydroxyflavanone	C_17_H_12_O_4_	[34]
	42	2(S)-6- methoxy-7-hydroxymethylene-4ʹ-hydroxyl-flavanone	C_17_H_16_O_5_	[20]
	43	corylifol F	C_17_H_14_O_5_	[35]
	44	corylifol G	C_25_H_26_O_5_	[35]
	45	bakuflavanone	C_20_H_20_O_5_	[32]
	46	isoneobavaisoflavone	C_20_H_18_O_4_	[26]
	47	corylifol A	C_25_H_26_O_4_	[26]
	48	erythrinin A	C_20_H_16_O_4_	[26]
	49	5,7,4ʹ-trihydroxyisoflavone	C_15_H_10_O_5_	[34]
	50	7-*O*-methylluteone	C_21_H_20_O_6_	[24]
	51	corylin	C_20_H_16_O_4_	[24]
	52	neobavaisoflavone	C_20_H_18_O_4_	[24]
	53	corylifols D	C_20_H_18_O_5_	[36]
	54	corylifols E	C_20_H_18_O_6_	[36]
	55	daidzin	C_21_H_20_O_9_	[37]
	56	daidzein	C_15_H_10_O_4_	[38]
	57	biochanin A	C_16_H_11_O_5_	[38]
	58	7-*O*-methylcorylifol A	C_26_H_28_O_4_	[39]
	59	7-*O*-Isoprenylneobavaisoflavone	C_25_H_26_O_4_	[39]
	60	7-*O*-Isoprenylcorylifol A	C_30_H_34_O_4_	[39]
	61	corylinin	C_25_H_26_O_4_	[40]
	62	neocorylin	C_25_H_24_O_4_	[41]
	63	8-prenyldaidzein	C_20_H_18_O_4_	[42]
	64	bavadin	C_27_H_30_O_13_	[43]
	65	bavarigenin	C_22_H_22_O_7_	[43]
	66	corylinal	C_16_H_10_O_5_	[44]
	67	corylinal methyl ether	C_17_H_12_O_5_	[45]
	68	psoralenol	C_20_H_18_O_5_	[46]
	69	7-Methoxybakuchiol	C_21_H_20_O_5_	[46]
	70	3″-acetoxy-7-methoxybakuchiol	C_23_H_22_O_6_	[46]
	71	3″,7-acetoxybakuchiol	C_24_H_22_O_7_	[46]
	72	wighteone	C_20_H_18_O_5_	[22]
	73	isowighteone	C_20_H_18_O_5_	[22]
	74	neo-bavaisoflavone	C_20_H_18_O_4_	[30]
	75	4ʹ,7-dihydroxy-3ʹ-(6″ β-hydroxy-3ʺ,7ʺ-dimethyl-,2ʺ,7ʺ-dibutenyl)-geranylisoflavone	C_25_H_26_O_5_	[20]
	76	4ʹ,7-dihydroxy-3ʹ-(7ʺ-hydroxy-7ʺ-methyl-2ʺ,5ʺ-dibutenyl)-geranylisoflavone	C_25_H_26_O_5_	[20]
	77	corylisoflavone A	C_18_H_18_O_6_	[47]
	78	bavachalcone	C_20_H_20_O_4_	[29]
	79	Isobavachalcone	C_20_H_20_O_4_	[29]
	80	Isoneobavachalcone	C_15_H_12_O_5_	[29]
	81	isobavachromene	C_20_H_18_O_4_	[48]
	82	Bavachromene	C_20_H_18_O_4_	[49]
	84	4,2ʹ-Dihydroxy-4ʹ-methoxy-5ʹ-(3″,3″-dimethylallyl)-chalcone	C_21_H_22_O_5_	[23]
	84	Bakuchalcone	C_20_H_20_O_5_	[50]
	85	brosimacutin G	C_20_H_20_O_6_	[26]
	86	corylifol B	C_20_H_20_O_5_	[26]
	87	Psorachalcone A	C_20_H_20_O_5_	[51]
	88	Psorachalcone B	C_20_H_20_O_5_	[51]
	89	bavachromanol	C_20_H_20_O_5_	[52]
	90	neobavachalcone	C_17_H_14_O_6_	[53]
	91	3,4- furanbavachalcone A	C_21_H_22_O_6_	[20]
	92	4,5- furanbavachalcone A	C_20_H_20_O_6_	[20]
	93	4,2ʹ-dihydroxy-2″ -(1‴-methylethyl)-2″-3″-dihydro-(4″,5″,3ʹ,4ʹ) furano chalkone	C_20_H_20_O_4_	[54]
	94	4ʹ-*O*-methylbavachalcone	C_21_H_22_O_4_	[54]
	95	xanthoangelol	C_25_H_28_O_4_	[22]
	96	Artonin ZA	C_20_H_18_O_4_	[35]
Monoterpene phenols	97	bakuchiol	C_18_H_24_O	[55]
	98	bisbakuchiols A	C_36_H_46_O_4_	[56]
	99	bisbakuchiols B	C_36_H_46_O_4_	[56]
	100	corylifolin	C_13_H_16_O	[57]
	101	bisbakuchiols C	C_36_H_48_O_3_	[58]
	102	cyclobakuchiol A	C_18_H_24_O	[59]
	103	cyclobakuchiol B	C_18_H_24_O	[59]
	104	2,3-epoxybakuchiol	C_18_H_24_O_2_	[59]
	105	Δ^1^,3-hydroxybakuchiol	C_18_H_24_O_2_	[59]
	106	Δ^3^,2-hydroxybakuchiol	C_18_H_24_O_2_	[59]
	107	cyclobakuchiol C	C_18_H_26_O_2_	[60]
	108	13-methoxyisobakuchiol	C_19_H_26_O_2_	[61]
	109	13-ethoxyisobakuchiol	C_20_H_28_O_2_	[61]
	110	12,13- dihydro-13-hydroxybakuchiol	C_18_H_26_O_2_	[61]
	111	Δ^10^-12,13-dihydro-12-(R)-methoxyisobakuchiol	C_19_H_26_O_2_	[61]
	112	Δ^10^-12,13-dihydro-12-(S)-methoxyisobakuchiol	C_19_H_26_O_2_	[61]
	113	15-demetyl-12,13-dihydro-13-ketobakuchiol	C_17_H_22_O_2_	[61]
	114	Δ^1,3^-bakuchiol	C_18_H_22_O	[61]
	115	12R,13-diolbakuchiol	C_18_H_26_O_3_	[24]
	116	12S,13-diolbakuchioll	C_18_H_26_O_3_	[24]
	117	psoracorylifol A	C_18_H_24_O_3_	[62]
	118	psoracorylifol B	C_18_H_24_O_3_	[62]
	119	psoracorylifol C	C_18_H_24_O_3_	[62]
	120	psoracorylifol D	C_18_H_24_O_2_	[62]
	121	psoracorylifol E	C_18_H_24_O_2_	[62]
	122	psoracorylifol F	C_18_H_24_O_2_	[63]
	123	psoralen ether	C_23_H_30_O	[64]
	124	psoracorylifol G	C_19_H_26_O	[64]
	125	psoracorylifol H	C_18_H_24_O_3_	[64]
	126	bisbakuchiol V	C_36_H_48_O_4_	[64]
	127	bisbakuchiol M	C_36_H_40_O_4_	[65]
	128	bisbakuchiol N	C_36_H_46_O_2_	[65]
	129	bisbakuchiol O	C_36_H_46_O_2_	[65]
	130	bisbakuchiol P	C_36_H_46_O_2_	[65]
	131	bisbakuchiol Q	C_36_H_46_O_3_	[65]
	132	bisbakuchiol R	C_37_H_50_O_4_	[65]
	133	bisbakuchiol S	C_37_H_50_O_4_	[65]
	134	bisbakuchiol T	C_36_H_46_O_3_	[65]
	135	bisbakuchiol U	C_36_H_46_O_3_	[65]
	136	bakuchiol ether A	C_29_H_42_O_2_	[65]
	137	bakuchiol ether B	C_33_H_50_O_2_	[65]
	138	bakuchiol ether C	C_32_H_46_O_2_	[65]
	139	Δ^11^-12-hydroxy-12-dimethyl bakuchiol	C_18_H_24_O_2_	[20]
Benzofurans	140	corylifonol	C_13_H_14_O_4_	[66]
	141	isocorylifonol	C_13_H_14_O_4_	[66]
	142	butylcnideoside A	C_21_H_26_O_9_	[67]
	143	dihydrobutylcnideoside A	C_21_H_28_O_9_	[67]
	144	isopsoralenoside butyl ester	C_21_H_26_O_9_	[67]
	145	dihydroisopsoralenoside butyl ester	C_21_H_28_O_9_	[67]
	146	isopsoralenoside methyl ester	C_18_H_20_O_9_	[67]
	147	dihydroisopsoralenoside methyl ester	C_18_H_22_O_9_	[67]
	148	psoralenoside	C_17_H_18_O_9_	[68]
	149	isopsoralenoside	C_17_H_18_O_9_	[68]
Other compounds	150	glycerol monoesters	C_21_H_44_O_3_	[69]
	151	glycerol diesters	C_27_H_52_O_5_	[69]
	152	triacylglycerols	C_39_H_74_O_6_	[69]
	153	raffinose	C_11_H_20_O_10_	[69]
	154	daucosterol	C_35_H_60_O_6_	[69]
	155	palmitic acid	C_16_H_32_O_2_	[70]
	156	stearic acid	C_18_H_36_O_2_	[70]
	157	linoleic acid	C_18_H_32_O_2_	[70]
	158	β-stigmasterol	C_29_H_48_O	[70]
	159	pinitol	C_7_H_14_O_6_	[71]
	160	uracil	C_4_H_4_N_2_O_2_	[37]
	161	p-hydroxybenzaldehyde	C_7_H_6_O_2_	[72]
	162	methyl p-hydroxybenzoate	C_8_H_8_O_3_	[16]
	163	psoralester	C_22_H_38_O_4_	[49]

### Coumarins

One of the primary pharmacological elements of PCL is coumarins, which exhibit a wide range of pharmacological effects, including anti-inflammatory, anticoagulant, anticancer, antibacterial, antiviral, antioxidant, neuroprotective and cardiovascular protection [73]. The most extensively researched coumarins are psoralen and isopsoralen, which are also the ingredients listed in the Chinese Pharmacopoeia as indicators for assessing the quality of PCL medicinal materials and decoction pieces. They have photosensitive activity and are effective treatments for vitiligo [74]. According to their structural properties, the 24 coumarin components that have been extracted and identified from PCL thus far are primarily categorized as furanocoumarins (1–7), coumestrols (8–19, 22, 24), pyranocoumarins (20) and other coumarins (21, 23). Table 2 (1–24) lists the specific compounds and Fig. 3 shows their chemical structures.

### Flavonoids

Flavonoids are one of the key active ingredients of PCL. So far, 72 flavonoids have been isolated and identified from PCL, mainly including flavonoids (25–27), flavonols (28, 29), dihydroflavonoids (30–43), isoflavones (44–77) and chalcones (78–96). The names of the specific compounds are shown in Table 2 (25–96), and the corresponding chemical structures are displayed in Fig. 4. According to certain pharmacological research, flavonoids exhibit pharmacological activities that include antioxidant, anti-inflammatory, antibacterial, antitumor, and hypoglycemia. It has been thoroughly explored that the antioxidant action in particular will be developed as an antioxidant for the prevention and treatment of many diseases brought on by oxidative stress. Some researchers believe flavonoids can be used as an index component for PCL quality control because of their potent biological activity [69]. UPLC-MS is the main method for qualitative and quantitative analysis of flavonoids [75].

### Monoterpene phenols

Monoterpene phenols are thought to be one of PCL’s distinctive and primary active components. Bakuchiol is the first discovered monoterpene phenol compound, which is considered as one of the effective and toxic components of PCL. Therefore, it is necessary to limit it as an indicator component [69]. Thus far, 43 monoterpene phenolic components have been isolated from PCL. The names of the specific compounds are shown in Table 2 (97–139), and the corresponding chemical structures are shown in Fig. 5.

### Benzofurans

Benzofurans are a group of heterocyclic compounds that are widely found in nature and have shown great medicinal value in terms of antibacterial, anti-inflammatory, anticancer, insecticidal and kinase inhibiting properties [76]. These compounds have been extensively studied by many scholars. To date, 10 benzofuran compounds have been isolated from PCL, including corylifonol (140), isocorylifonol (141) [66], butylcnideoside A (142), dihydrobutylcnideoside A (143), isopsoralenoside butyl ester (144), dihydroisopsoralenoside butyl ester (145), isopsoralenoside methyl ester (146), dihydroisopsoralenoside methyl ester (147) [67], psoralenoside (148), isopsoralenoside (149) [68]. The chemical structures are shown in Fig. 6. However, we found that there are currently fewer studies on the pharmacological activity of benzofurans, thus future study should focus more on this area.

### Other compounds

In addition to the aforementioned primary components, PCL also contains a wide range of additional chemical substances, such as lipid components like glycerol monoesters (150), glycerol diesters (151), triacylglycerols (152). Polysaccharide component like raffinose (153). Glycoside component like daucosterol (154) [69]. Fatty acid components like palmitic acid (155), stearic acid (156) and linoleic acid (157) [70]. In addition, it also contains pinitol (159) [71], β-stigmasterol (158) [70], uracil (160) [37], p-hydroxybenzaldehyde (161) [72], methyl p-hydroxybenzoate (162) [16] and psoralester (163) [49]. Potassium, manganese, calcium, iron, copper, zinc, antimony, rubidium, strontium, selenium, and other trace elements [69]. The chemical structures are shown in Fig. 7.

## Pharmacological effects

**Fig. 8:**
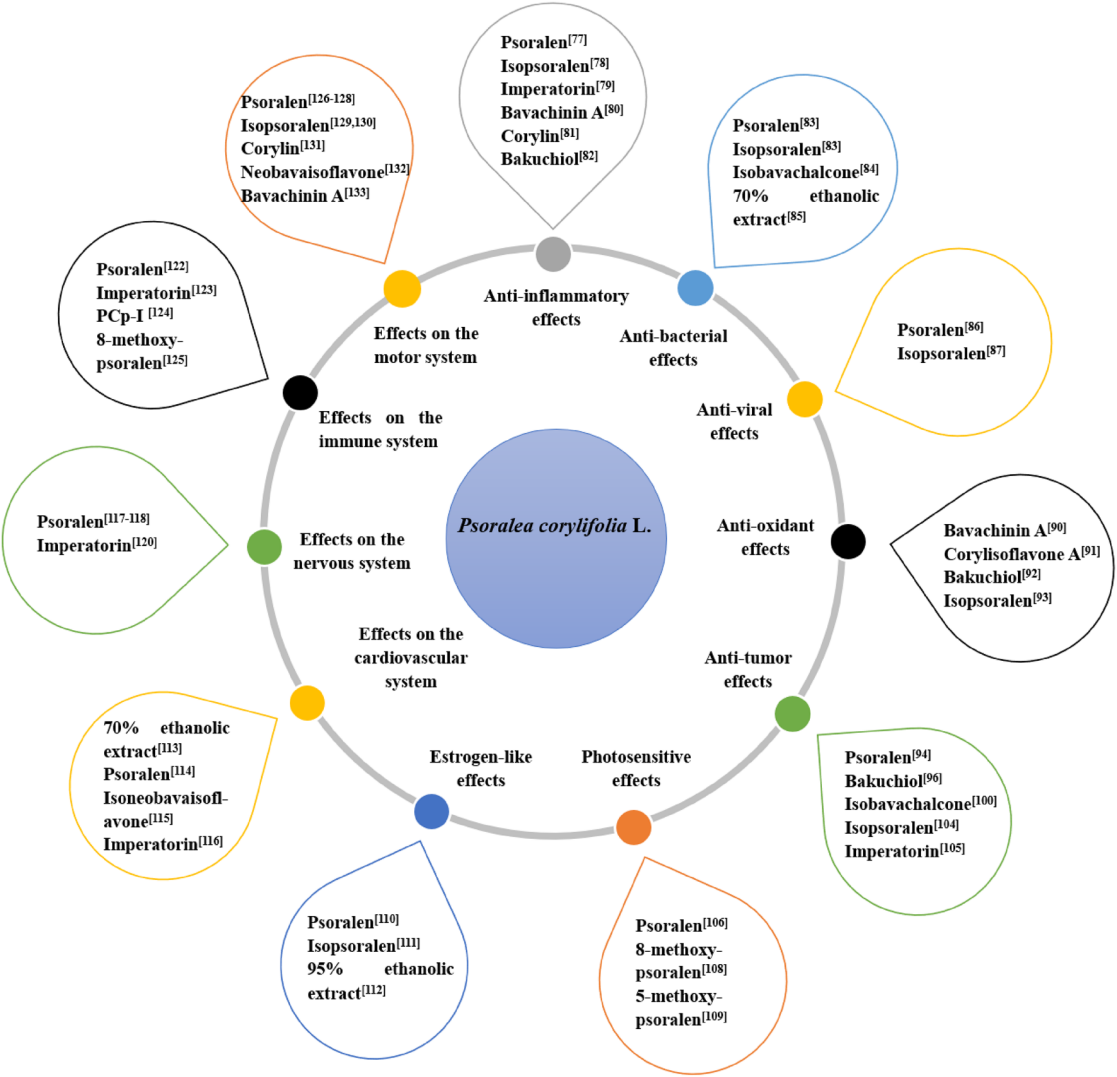
The relationship between pharmacology and main chemical constituents of PCL

Modern pharmacological studies have shown that fresh PCL exhibits anti-inflammatory, antibacterial, antiviral, antioxidative, anticancer, estrogen-like action, and photosensitivity. Additionally, it demonstrates extraordinary medical efficacy in controlling the actions of the immune system, nervous system, motor system, and cardiovascular system. These pharmacological effects are all covered in the sections that follow. Pharmacological activities of PCL and its active compounds are shown in Table 3 and Fig. 8.

**Table 3:** Pharmacological effects of PCL

Pharmacological effects	Active extract/fraction/compounds	Mechanism	Effective dose	Material or mode	Study design	References
Anti-inflammatory effects	Psoralen	Reduced the release of inflammatory factors and inflammatory cell infiltration	5, 10, 20 mg/kg	Dextran sulfate sodium-induced ulcerative colitis mice	In vivo	[77]
	Isopsoralen	Targeted MIF and reduced the release of inflammatory factors	10, 20 μM; 5, 20 mg/kg	Fibroblast-like synoviocytes; Collagen-induced arthritis mice	In vitro; In vivo	[78]
	Imperatorin	Inhibited COX-2, iNOS, and NF-κB activity and reduced circulating cytokines	ED50 = 4.53 mg/kg	Acetic acid and formalin -induced mice	In vivo	[79]
	Bavachinin A	Suppressed phosphorylation of p38 and JNK and inhibited expression and secretion of inflammatory factors in macrophages	6.25, 12.5, 25 μM	RAW264.7 cells stimulated by LPS	In vitro	[80]
	Corylin	Inhibited pro-caspase-1 self-shear activation	20 μM	NLRP3, NLRC4, AIM2 inflammasomes with immortalized bone marrow-derived macrophages	In vitro	[81]
	Bakuchiol	Activated SIRT1 signaling pathway	1, 5, 10 μM	LPS-induced cardiomyocyte inflammation myocytes	In vitro	[82]
Antibacterial effects	Psoralen	Inhibited the growth of biofilms	MIC = 6.25 μg/mL	Planktonic porphyromonas gingivalis and porphyromonas gingivalis	In vitro	[83]
	Isopsoralen	Inhibited the growth of biofilms	MIC = 3.125 μg/mL	Planktonic porphyromonas gingivalis and porphyromonas gingivalis	In vitro	[83]
	Isobavachalcone	Changed the bacterial morphology, destroy the cell membrane, increased the permeability of cell wall and the amount of soluble protein leakage in the cell	MIC = 0.078 mg/mL	Staphylococcus aureus, escherichia coli and pseudomonas aeruginosa	In vitro	[84]
Antiviral effects	Isopsoralen	Inhibited the lytic replication of the virus	IC50 = 28.95 μM	Murine gammaherpesvirus 68	In vitro	[86]
	Psoralen	Increased the neutralizing antibody titer of each dengue serotype	50 μg/mL	Mice and nonhuman primates	In vivo	[87]
	Psoralen	Down-regulated the expression of FOXO1 replication	IC50 = 63.7 ​ ± ​14.53 μm	HepG2.2.15 ​cells	In vitro	[88]
Antioxidant effects	Bavachinin A	Increased the activity of antioxidant enzymes and mitochondrial membrane potential	10^–6^ M	Aβ-induced PC12 cells	In vitro	[90]
	Corylisoflavone A	Reduced ROS levels through mitochondrial and non-mitochondrial pathways and regulated Nrf2 signaling pathway	10^–3^, 10^–2^, 10^–1^ μM	UVB-induced HaCaT cells	In vitro	[91]
	Bakuchiol	Activated the SIRT3/SOD2 signaling pathway	20 mg/kg	Doxorubicin-induced cardiotoxicity in mice	In vivo	[92]
	Isopsoralen	Regulated the PPAR-γ/Wnt pathway	10 mg/kg	Ovariectomized rats	In vivo	[93]
Anticancereffects						
Block cell cycle	Psoralen	Inhibited Wnt/β-catenin pathway	IC10 = 8 μg/mL; IC10 = 12 μg/mL	MCF-7 cells; MDA-MB-231 cells	In vitro	[94]
	Psoralen	Blocked the G0/G1 and G2/M phases	10, 30, 50 and 100 μg/ml	PC3 cells	In vitro	[95]
	Bakuchiol	Induced S-phase arrest through the p38-ROS-p53 pathway and inhibited cell proliferation through the JNK pathway	30 μmol/L	MCF-7 cells		[96]
Induction of apoptosis	Psoralen	Induced apoptosis via caspase-3, p53, Bax, and Bcl-2 pathway	10, 30, 50 and 100 μg/mL	SMMC-7721 cells	In vitro	[97]
	Psoralen	Depolymerized the cytoskeletal protein F-actin	25, 30, 50 μg/mL	BGC-803 cells	In vitro	[98]
	Psoralen	Triggered endoplasmic reticulum stress	10, 20, 40 and 80 μM	SMMC-7721 cells	In vitro	[99]
	Isobavachalcone	Regulated Sirtuins-related signaling pathway	60 μM	H460 cell	In vitro	[100]
	Isobavachalcone	Promoted the phosphorylation of AKT and its downstream GSK-3β, activated autophagy	10, 12, 14 μM	SW480cells	In vitro	[101]
	Isobavachalcone	Inhibited RSK2 activity and regulated the expression of c-Jun and Bcl-2/Bax	20, 40 μM	CNE-2Z cells	In vitro	[102]
Inhibit cell migration and invasion	Psoralen	Suppressed the expression levels of MKI67, PCNA, MMP2 and MMP9	30 μg/ml	HTB-47 and CRL-1932 cells	In vitro	[103]
	Isopsoralen	Up-regulated the expression of LncRNA THOR and down-regulated the expression of miR-153-5p	10、30、90 μg/ml	Tu686 cells	In vitro	[104]
	Imperatorin	Downregulated the expression of HMGB2	25, 50 µM	SKBR3 cells	In vitro	[105]
Photosensitive effects	Psoralen	Inhibited cell proliferation, reducted DNA synthesis and inducted inflammatory cell apoptosis	0.4 mg/kg	Patients with psoriasis	In vivo	[106]
	Psoralen	Normalized the expression of abnormal NGF and Sema3A in the epidermis	0.4 mg/kg	Patients with atopic dermatitis	In vivo	[107]
Estrogen-like effects	Psoralen	Bind to ERα and inhibited the activation of TLR4-IRAK4-NF-κB pathway	3.125, 6.25, 12.5 μg/mL	Human periodontalligament cells	In vitro	[110]
	Isopsoralen	Activated ERα and regulated the PI3K/AKT pathway	5, 10 mg/kg	Mice with spinal cord injury	In vivo	[111]
Regulation of cardiovascular system functions	70% ethanol extracts of PCL	Induced vasodilation through endothelium-dependent and non-dependent pathways	10–600 μg/mL	Rat aortic rings	In vitro	[113]
	Psoralen	Exerted vasodilatory effects via endothelium-dependent NO pathway and promoted eNOS protein expression	0.1, 1, 10 μM	Rat thoracic aortas	In vitro	[114]
	Bakuchiol	Exerted vasodilatory effects via non-endothelium dependent pathway of opening inwardly rectifying potassium channel	0.1, 1, 10 μM	Rat thoracic aortas	In vitro	[114]
	Isobavachalcone	Promoted HUVEC migration, reduced the secretion of inflammatory factors, and down-regulated NOD1/RIP2 signaling pathway.resistance	2.5, 5, 10 μM	LPS-induced HUVEC cells	In vitro	[115]
Regulation of nervous system functions	70% ethanol extracts of PCL	Improved the learning and memory ability by increasing the expressions of ERα, ERβ, FSHR and LHR	0.5 g/kg	APP/PS1mice	In vivo	[117]
	70% ethanol extracts of PCL	Increased the content of 5-hydroxytryptamine in serum and hippocampus	15, 30, 60 mg/kg	Mice	In vivo	[118]
	Psoralen	Inhibited neural stem cell proliferation and promoted stem cell differentiation	10, 50, 100 nM	Adult neural stem cells	In vitro	[119]
	Psoralen	Reduced the expression of APP, BACE1 and Aβ protein through the ERβ-mediated P-ERK pathway	1 μM	Aβ-induced PC12 cells	In vitro	[120]
	Imperatorin	Mitigated proinflammatory cytokines, oxidative stress and modulated brain-derived neurotropic factor	5, 10 mg/kg	LPS-induced mice	In vivo	[121]
Regulation of immune system functions	Psoralen	Regulated Th1/Th2 cell balance and inhibited the release of inflammatory factors TNF-α, IL-6 and IL-1β	40 mg/kg	Type II collagen-induced rheumatoid arthritis in mice	In vivo	[123]
	Imperatorin	Suppressed anaphylactic reaction and IgE-mediated allergic responses by inhibiting multiple steps of FceRI Signaling in Mast Cell	15, 30, 60 mg/kg	Passive cutaneous anaphylaxis in mice	In vivo	[124]
	PCp-I (a polysaccharide from PCL)	Activated RAW264.7 cells through NF-κB/MAPK signalling pathway	100, 200, 400 and 800 μg/m L	RAW264.7 cells	In vitro	[125]
Regulation of motor system functions	Psoralen	Promote osteoclast bone resorption and osteoblast bone differentiation through the ERK pathway	2.5, 5, 10, 20 and 40 μM	BMM cells and MC3T3-E1 cells	In vitro	[127]
	Psoralen	Increased the expression of PPARγ, osteocalcin, and trabecular bone area	35 mg/kg	Rabbits with steroid-induced avascular necrosis of the femoral head	In vivo	[128]
	Psoralen	Promoted the proliferation of bone marrow mesenchymal stem cells and induced bone marrow mesenchymal stem cells to differentiate into cartilage	10 μM	Rat bone marrow mesenchymal stem cells	In vitro	[129]
	Isopsoralen	Inhibited the Axin2/PPAR-γ signaling pathway and activating the Wnt signaling pathway	25 mg/kg	Dexamethasone sodium injection-induced in rats	In vivo	[130]
	Isopsoralen	Inhibited RANKL-induced osteoclast differentiation by inhibiting the NF-κB signaling pathway	30 μM	Primary mouse mononuclear macrophages	In vitro	[131]
	Neobavaisoflavone	Interfered the activation of key signaling pathways mediated by RANKL/RANK	30 mg/kg; 2, 4, 8 μM	Ovariectomized in mice; RAW264.7 cells and BMMC cells	In vivo; In vitro	[132]

## Anti-inflammatory effects

PCL has anti-inflammatory effect, which is closely related to regulating the inflammatory signal pathway, preventing the production and release of inflammatory factors, and reducing the infiltration of inflammatory cells. Coumarins, flavonoids and monoterpene phenols are considered to be the main pharmacodynamic substances. In a study, it was discovered that psoralen (5, 10, 20 mg/kg), a coumarin component isolated from PCL, could, in a dose-dependent manner, lower the expression of IL-6, IL-1, and TNF-inflammatory factors in serum and colonic tissues of mice with ulcerative colitis and reduce inflammatory cell infiltration, and had a positive therapeutic effect on the ulcerative colitis in mice that was brought on by dextran sodium sulfate [77]. In the treatment of rheumatoid arthritis, isopsoralen (in vitro: 10, 20 μM; in vivo: 5, 20 mg/kg) could improve inflammatory response by targeting macrophage migration inhibitory factor (MIF) and reducing the release of inflammatory factors [78]. In another anti-inflammatory study, imperatorin (ED50 = 4.53 mg/kg) was found to have anti-inflammatory and analgesic effects by inhibiting the activities of COX-2, iNOS, and NF-B, as well as reducing circulating cytokines. This allowed it to effectively inhibit mice carrageenan-induced paw edema and acetic acid-induced torsion [79]. Bavachinin A is a flavonoid component from PCL, it (6.25, 12.5, 25 μM) could significantly reduce liver inflammation by suppressing the phosphorylation of p38 and JNK, and reducing the secretion and expression of inflammatory factors in macrophages [80]. Corylin (20 μM) could inhibit the activation of inflammatory bodies of NLRP3, NLRC4 and AIM2, and further reduce the immune inflammatory response mediated by inflammatory bodies, which may be related to the inhibition of self-shearing activation of Caspase-1 [81]. Bakuchiol (1, 5, 10 μM), a monoterpene phenol component of PCL, could effectively inhibit the release of inflammatory factors IL-1and TNF-α, increase the protein levels of SIRT1 and Bcl-2, and decrease the protein expression of Bax by activating SIRT1 signaling pathway, thereby alleviating LPS-induced myocardial inflammation and cardiomyocyte apoptosis [82].

## Antibacterial effects

According to a study, psoralen and isopsoralen had potent antibacterial properties since they could stop the growth of planktonic porphyromonas gingivalis and porphyromonas gingivalis biofilms [83]. Zhou et al. [84] found that isobavachalcone (Minimum inhibitory concentration (MIC) = 0.078 mg/mL) significantly inhibited the activity of methicillin-resistant staphylococcus aureus, extended spectrum -lactamases staphylococcus aureus, and staphylococcus aureus, and its mechanism may involve altering bacterial morphology, destroying the cell membrane, increasing the permeability of cell wall and the amount of soluble protein leakage in the cell. Another study examined the antibacterial activity of 70% ethanol extracts of PCL, and it found that the main antibacterial active substances against valsa mali were bakuchiol, bakuchicin, bavachalcone, psoralidin, and bavachin, with the EC50 values for the five compounds being 6.117, 60.441, 3.420, 36.815, and 6.144 mg/L, respectively [85]. New discoveries of the biological functions of these natural compounds are of positive significance to the development of new and more effective botanical fungicides.

## Antiviral effects

In the prevention and treatment of viruses, PCL has a wide range of applications. In a study, isopsoralen (IC50 = 28.95 μM) successfully inhibited the lytic replication of murine gamma herpesvirus 68 (MHV-68) in a dose-dependent manner [86]. In mice and nonhuman primates, the psoralen-inactivated dengue virus vaccine was immunogenic, according to research by Sundaram et al. [87], and this led to a significant increase in the titer of neutralizing antibodies to each dengue serotype. Further study revealed that, in contrast to conventional virus inactivation, psoralen inactivated pathogens at the nucleic acid level, rendered the virus noninfectious while maintained the integrity of virus particles and RNA, potentially protected envelope protein epitopes crucial for protective antiviral immune responses. Another study found that psoralen (CC50 = 413.5 ± 12.98 μM) might suppress the transcription of the hepatitis B virus (HBV) RNA by down-regulating FOXO1 expression, which reduced HBV replication [88]. In addition, psoralen could effectively render transfusion-transmissible viruses like the human immunodeficiency virus, hepatitis B virus, hepatitis C virus, and severe acute respiratory syndrome coronavirus (SARS-CoV) inactive, which was very useful for lowering the risk of infection with a human plasma-derived virus [89].

## Antioxidant effects

Failure to eliminate free radicals quickly in the oxidation state will harm lipids, proteins, nucleic acids, and other molecules, interfere with a number of biological functions, and eventually result in disease. Thus, it is crucial to develop natural compounds that have antioxidant action. Yang et al. [90] evaluated the antioxidant effect of bavachinin A (10^–6^ M) and found that it protected nerve cells from oxidative damage by reducing the content of MDA in cells, increasing the activity of antioxidant enzymes SOD and GSH-Px, and improving mitochondrial membrane potential. Another study demonstrated the protective effects of corylisoflavone A (10^–3^, 10^–2^, 10^–1^ μM) against UVB-induced oxidative damage in HaCaT cells. This effect may be mediated by lowering ROS levels through mitochondrial and non-mitochondrial pathways and modulating the Nrf2 signaling pathway [91]. An in vivo study discovered that bakuchiol (20 mg/kg) might reduce doxorubicin-induced cardiotoxicity and safeguard heart function by activating the SIRT3/SOD2 signaling pathway and preventing myocardial oxidative stress injury and apoptosis [92]. According to Zhang’s research [93], isopsoralen (10 mg/kg) could inhibit oxidative stress-mediated osteoporosis via regulating the PPAR-γ/Wnt pathway. Isopsoralen has potential applicability as a treatment for osteoporosis brought on by oxidative stress, however some of the mechanisms of action require more research.

## Anticancer effects

Cancer is a public health problem of global concern, and its morbidity and mortality are increasing year by year, seriously affecting human life and health. A growing number of studies have shown that Chinese herbal medicine possesses potent anticancer properties with low side effects, so it is an ideal anticancer drug candidate. PCL has been reported to have anticancer effect in numerous studies, and the main anticancer mechanisms are blocking cell cycle, inducing cell apoptosis, inhibiting cell migration and invasion.

### Block cell cycle

An in vitro study found that psoralen dramatically reduced cell proliferation by inducing G0/G1 arrest in MCF-7 cells and G2/M phase arrest in MDA-MB-231 cells through the inhibition of Wnt/-catenin pathway activity [94]. In addition, psoralen could inhibit the proliferation of prostate cancer cells in a time- (24 h, 48 h, 72 h, 96 h) and dose-dependent (10, 30, 50, 100 μg/ml) manner, and its mechanism involved preventing the G0/G1 and G2/M phases [95]. Zhang et al. [96] showed that bakuchiol could induce S-phase arrest in MCF-7 cells through the p38-ROS-p53 pathway and inhibit cell proliferation through the JNK pathway, which laid the groundwork for further research and clinical use of bakuchiol against breast cancer.

### Induction of apoptosis

Apoptosis is primarily initiated through the death receptor pathway, endoplasmic reticulum pathway and mitochondrial pathway. Studies found that psoralen could continuously trigger the endoplasmic reticulum stress response, upregulate the protein expression levels of caspase-3, p53 and Bax, and downregulate the levels of Bcl-2, leading to hepatoma cell apoptosis. Besides, psoralen could depolymerize the cytoskeleton protein F-actin, which prevented stomach cancer cells from proliferating and induced apoptosis [97–99]. In human non-small cell lung cancer H460 cells, isobavachalcone was shown by Ren et al. [100] to induce apoptosis by increasing the generation of ROS, and its molecular mechanism may be connected to influencing intracellular Sirtuins-related signaling pathways. Isobavachalcone also had the potential to stimulate autophagy, phosphorylate AKT and its downstream GSK-3, and finally induced death in colorectal cancer cells [101]. Additionally, isobavachalcone had the ability to drastically reduce the growth of nasopharyngeal cancer CNE-2Z cells and induce apoptosis, and this effect may be caused by reducing RSK2 activity and regulating the expression of c-Jun and Bcl-2/Bax [102]. According to these findings, the application of isobavachalcone in the treatment of lung cancer, colorectal cancer, and nasopharyngeal cancer may be a promising adjuvant chemotherapy.

### Inhibit cell migration and invasion

Important biological traits of malignant tumors include invasion and migration. To effectively manage the cancer process and increase patient longevity, tumor cell migration and invasion must be effectively inhibited. A study found that psoralen could slow the progression of kidney cancer by inhibiting the expression of MKI67, PCNA, MMP2 and MMP9 as well as the proliferation, invasion, and migration of kidney cancer cells HTB-47 and CRL-1932 [103]. Another investigation revealed that isopsoralen could significantly reduce the proliferative, migratory, and invading abilities of laryngeal cancer cells Tu686, possibly by upregulating the expression of LncRNA THOR and downregulating the expression of miR-153-5p [104]. Gao et al. [105] demonstrated that imperatorin could inhibit the invasion and migration of breast cancer cells by downregulating the expression of high mobility group box B2 (HMGB2), offering new insight into the underlying mechanism of imperatorin’s action in human breast cancer. Further research is necessary to determine how imperatorin reduce the expression of HMGB2 in breast cancer cells.

In summary, PCL can block cell cycle, induce cell apoptosis, and inhibit cell migration and invasion to prevent tumor growth. However, we have noticed that many studies have concentrated on cells in vitro. Future research should completely examine PCL’s anti-tumor mechanism in conjunction with animal and clinical investigations, and the anticancer effects of PCL’s should be fully utilized for medical value.

## Photosensitive effects

According to a study, psoralen combined with UVA phototherapy (PUVA) could effectively treat inflammatory skin diseases, and the mechanism of action may be related to the suppression of cell proliferation, reduction of DNA synthesis, and activation of inflammatory cell apoptosis [106]. Treatment with PUVA for atopic dermatitis (AD) patients was able to minimize the over innervation of the epidermis and normalize the production of aberrant nerve growth factor (NGF) and signaling protein 3A (Sema3A) in the epidermis. Additionally, the benefits of PUVA therapy were low frequency, low dose, and high efficacy, which might be popularized in clinical settings [107]. Both 8-methoxy-psoralen (8-MOP) and 5-methoxy-psoralen (5-MOP) have photosensitive activity, however 5-MOP has a lower rate of adverse events and photosensitive activity overall. Psoriasis, vitiligo, and mycosis fungoides can all be effectively treated with oral or local administration combined with UVA. [108, 109].

## Estrogen-like effects

Although estrogen is crucial for female development and several disease processes, prolonged estrogen use can have a number of side effects. As a result, phytoestrogens with milder effects, which have become a hot research topic, have been sought as alternatives to estrogen. In studies, PCL is found to have estrogen-like effects. Psoralen (3.125, 6.25, 12.5 μg/mL) could bind to estrogen receptor α (ERα) and inhibit the activation of TLR4-IRAK4-NF-κB pathway, exerting an anti-inflammatory effect via estrogen-like action, and this effect could be reversed by estrogen receptor inhibitors [110]. Another study found that isopsoralen (5, 10 mg/kg) activated ER and regulated the PI3K/AKT pathway to exert estrogen-like neuroprotection against spinal cord injury-induced apoptosis [111]. An in vivo investigation revealed that different polar parts of the 95% ethanol extracts of PCL all exhibited estrogen-like activity and could greatly speed up the growth of mice’s uterine coefficient and body weight. By comparative analysis, the petroleum ether part had the highest activity, which may be related to the part’s high bakuchiol content [112].

## Effects on the cardiovascular system

Coronary heart disease, hypertension, hyperlipidemia, angina pectoris, arrhythmia, etc. are examples of cardiovascular diseases. Effective cardiovascular disease prevention and treatment is a crucial problem because it is one of the diseases with the highest morbidity and mortality rates in the world. Some studies have shown that PCL and its active components are effective in both preventing and treating cardiovascular disorders. Kassahun et al. [113] found that the 70% ethanol extracts of PCL (PCE) could induce arterial dilatation in rats through endothelium-dependent and non-dependent pathways. Further studies revealed that the active components of PCE were bakuchiol, isobavachalcone, isopsoralen, and psoralen. At concentrations between 10 and 600 μg/mL, these compounds inhibited TRPC3 channels, altered TRPC3-mediated ionic currents, and reduced the vasoconstrictive effects of phenylalanine. A study found that psoralen and bakuchiol played a role in vasodilation through endothelium-dependent NO pathway and promoting the expression of eNOS protein in endothelial cells. In addition, bakuchiol may open an inwardly rectifying potassium channel via a non-endothelium dependent pathway, which has significant clinical implications for controlling blood pressure and protecting the cardiovascular system [114]. Another study found that isoneobavaisoflavone could significantly increase the ability of human umbilical vein endothelial cells (HUVECs) to migrate, decrease the secretion of inflammatory factors, and inhibit the NOD1/RIP2 signaling pathway, thereby inhibiting the inflammatory response of vascular endothelial cells. This finding suggests that isoneobavaisoflavone may be used to prevent the development of atherosclerosis, diabetic vasculopathy and other conditions that are intimately linked to the inflammatory response of blood vessels [115]. Imperatorin, another active component in PCL, could lower the occurrence of cardiovascular disorders such as hypertension, hyperlipidemia, and heart failure by inhibiting myocardial remodeling, lowering myocardial weight, and reducing fibrosis surrounding the myocardium or micro-vessels [116].

## Effects on the nervous system

PCL and its active components can regulate the central nervous system, which has considerable promise for treating diseases of the central nervous system. It was discovered that 70% ethanol extracts of PCL (0.5 g/kg) could significantly improve spatial learning memory impairment in APP/PS1 mice, and its mechanism may be related to the increased expression of ER α, ER β, follicle-stimulating hormone receptor (FSHR) and luteinizing hormone receptor (LHR), which is expected to become a new drug against Alzheimer’s disease [117]. In addition, 70% ethanol extracts of PCL could exert antidepressant effects by increasing the content of 5-hydroxytryptamine levels in serum and hippocampus [118]. According to Ning et al. [119], psoralen (10, 50, 100 nM) had good potential therapeutic effects on central nervous system injury and neurodegenerative diseases because it could effectively regulate the specific gene expression profile of neural stem cells, inhibit their proliferation, and promote their differentiation into astrocytes. Another study found that psoralen could suppress the progression of Alzheimer’s disease by reducing the expression of amyloid precursor, β-secretase (BACE1), and Aβ protein, as well as Aβ deposition and its toxic effects on neurons through the estrogen receptor β (ERβ)-mediated P-ERK pathway [120]. Chowdhury et al. [121] showed that imperatorin (5, 10 mg/kg) pre-treatment significantly decreased levels of the pro-inflammatory cytokines TNF-α and IL-6, increased levels of brain-derived neurotrophic factor (BDNF), and reduced levels of oxidative stress and acetylcholinesterase (AChE) in the hippocampus and cerebral cortex of mice, reversing LPS-mediated cognitive impairment and neuroinflammation. In neurodegenerative illnesses like Alzheimer’s, imperatorin may be utilized as an adjunctive therapy. To pinpoint the precise mechanism of imperatorin’s neuroprotective properties, further thorough research is required.

## Effects on the immune system

Modern studies have demonstrated that PCL regulates immune function, which is specifically reflected in the regulation of immune organs, immune cells, immune molecules, hypersensitivity reactions, tumors, and transplant rejection, among other things. This explains the traditional effectiveness of PCL on kidney tonifying from the perspective of modern pharmacology [122]. Zhang et al. [123] investigated the effect of psoralen on Type II collagen-induced rat rheumatoid arthritis (RA), and found that psoralen could play an immunomodulatory role by regulating Th1/Th2 cell balance and inhibiting the release of TNF-α, IL-6 and IL-1β inflammatory factors, which would slow the progression of RA. Another study discovered that imperatorin (15, 30, 60 mg/kg) could lessen allergic reactions by decreasing proinflammatory cytokine expression in mast cells and inhibiting mast cell degranulation through several signaling pathways, including PI3K/Akt, MAPK, NF-B, and Nrf2/HO-1. This suggests that imperatorin is a potential therapeutic drug for allergic diseases [124]. A study on PCp-I, a polysaccharide derived from PCL, found that it could activate macrophages via the TLR4-mediated MAPK and NF-B signaling pathways, promote the release of NO, TNF-, and IL-6, and upregulate the expression levels of p65, p38, ERK, and JNK proteins and genes, acting as an immunomodulator on RAW264.7 cells. Therefore, PCp-I has the potential to be developed as inherent immune response regulators [125]. Another study revealed that 8-MOP combined with UVA light irradiation could be used to treat organ transplant rejection and lower the risk of infection, and its mechanism may involve initiating programmed lymphocyte death, stimulating the maturation of immature dendritic cells, promoting T-regulatory cell expansion and anti-inflammatory cytokine expression, but the specific mechanism of action remains to be further investigated [126].

## Effects on the motor system

PCL has been widely used in the prescription of tonifying kidney and strengthening bone. Modern pharmacological studies have shown that psoralea have pharmacological activities in the prevention of osteoporosis, the promotion of fracture healing, and the regeneration of articular cartilage. A study found that psoralen could speed up fracture healing by promoting osteoclast bone resorption and osteoblast bone differentiation through the ERK pathway [127]. Besides, psoralen could play a positive role in the treatment of hormonal ischemia necrosis of the femoral head by decreasing PPAR expression in hormonal head necrosis and boosting osteocalcin expression, reducing bone marrow cell lipogenesis, and promoting calcium deposition [128]. Another study showed that the combination of psoralen and transforming growth factor β1 (TGF-1) could stimulate bone marrow mesenchymal stem cell proliferation and induce differentiation of bone marrow mesenchymal stem cells into chondrocytes, which could be used as a novel method of articular cartilage repair [129]. Wang et al. [130] reported that isopsoralen at a dose of 25 mg/kg could improve bone metabolism abnormalities and promote bone formation in rats by blocking the Axin2/PPAR-signaling pathway and activating the Wnt signaling pathway. In addition, isopsoralen could prevent the RANKL-induced differentiation of osteoclasts by inhibiting the NF-κB signaling pathway [131]. Other studies revealed that the flavonoids corylin, neobavaisoflavone and bavachinin A in PCL could all inhibit osteoclast differentiation and function, significantly slow down the bone loss brought on by ovariectomizing, and effectively treat post-menopausal osteoporosis. Their molecular mechanisms may be related to the inhibition of NF-B, MAPK, and PI3k/AKT signaling pathway activation [132–134]. These experiments provide a fresh idea for the development of new osteoporosis medications.

## Other activities

In addition to the pharmacological actions listed above, PCL and its active components also have the effects of enhancing reproductive abilities, improving insulin resistance, inhibiting scar formation, reducing pigmentation, anti-skin photoaging and anti-fibrosis. According to Wan et al. [135], psoralen could considerably up-regulate the expression levels of the genes OCT-4, Stra8, SCP3, and Itgb1, which provided preliminary evidence that psoralen may cause mesenchymal stem cells to differentiate into male germ cells. However, in vivo testing is necessary to confirm whether psoralen can indeed increase male fertility. In another study, it was shown that isobavachromene could enhance adipocyte glucose uptake by activating the PI3K/Akt signaling pathway and improve adipocyte inflammation by inhibiting JNK, NF-κB and SOCS-3 inflammatory signaling pathways, thus improving insulin resistance in 3T3-L1 adipocytes. This study provides a new way for the treatment of type 2 diabetes [136]. A study conducted in vitro revealed that psoralen could suppress the SMA protein through the TGF-β1/Smurf2 signaling pathway, lowering the expression of type I collagen and preventing the formation of scars [137]. Additionally, psoralen, isopsoralen, corylin all demonstrated anti-photoaging effects on skin, and their therapeutic mechanisms involved reducing inflammation, boosting antioxidant defenses, preventing apoptosis, and enhancing the production of collagen. These results provide new insights into the clinical application of PCL for the prevention and treatment of photoaging [91, 138, 139]. Other studies showed that psoralen could inhibit MITF, TYR, TRP-1, and TRP-2 expression levels as well as tyrosinase activity and melanin synthesis through the ER/MAPK and p38-MAPK signaling pathways. This information is extremely important for the clinical treatment of melasma and other skin pigmentation disorders [140, 141]. In addition, psoralen was effective in treating bleomycin-induced idiopathic pulmonary fibrosis, and its mechanism may involve preventing fibroblast activation, collagen deposition, and the release of inflammatory factors [142].

## Toxicology

**Fig. 9:**
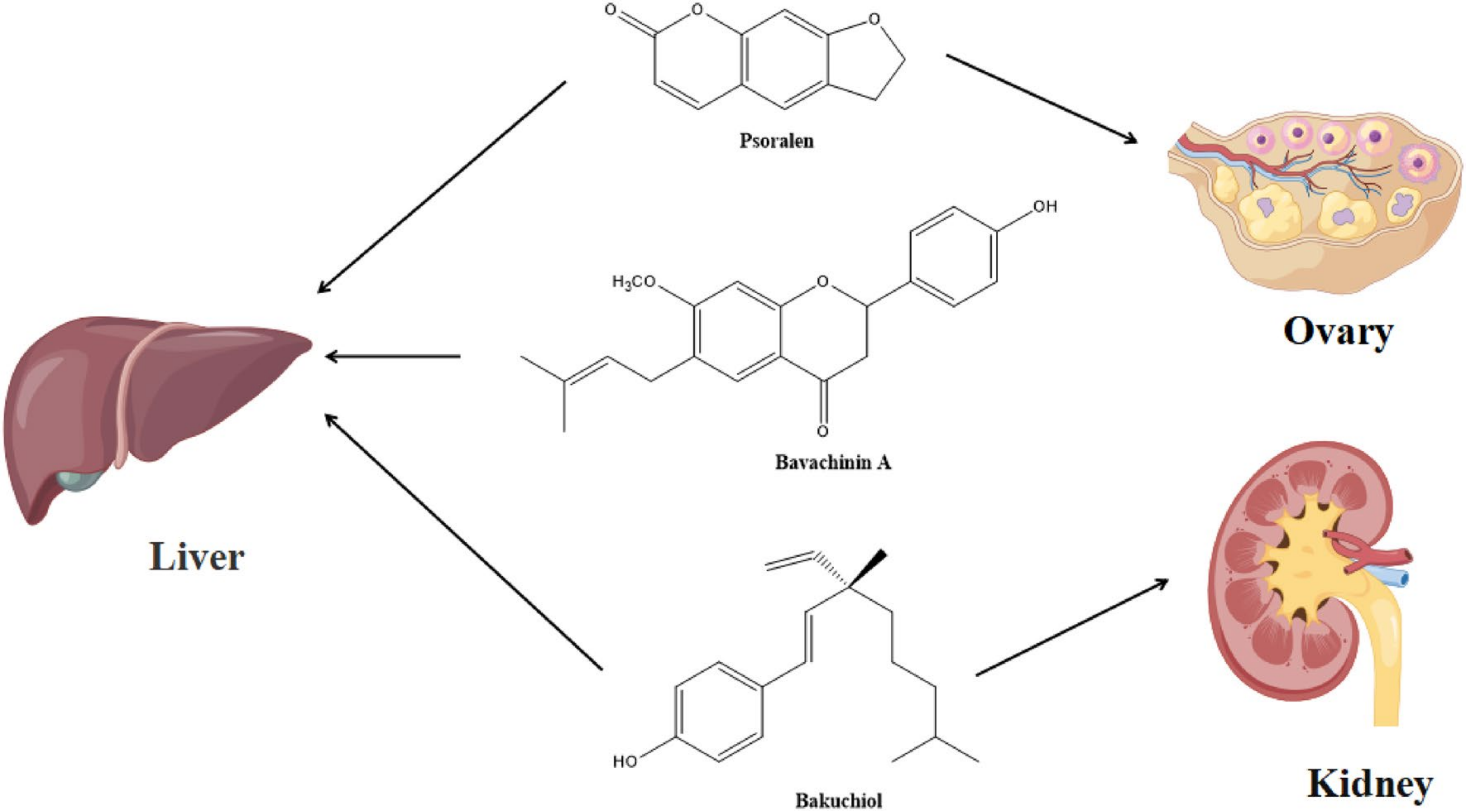
The main toxic components of psoralea and their action sites

According to “Lei’s Treatise on Preparing Drugs”, PCL is poisonous, while other medical books do not mention its toxicity. With the in-depth investigation of fresh PCL, increasing amounts of evidence have demonstrated that the chronic or excessive administration of PCL and its active constituents can cause hepatotoxicity, nephrotoxicity, phototoxicity, developmental toxicity and reproductive toxicity. Hepatotoxicity is the most frequently reported of them all. It is crucial to understand the mechanism and material foundation of PCL’s toxicity in order to effectively manage PCL’s toxicity and guarantee clinical safety. We provide a comprehensive overview of current studies on the toxicology of PCL in this section. Table 4 and Fig. 9 illustrate PCL’s and its active components’ toxicity.

**Table 4:** The toxicity of PCL

Toxicology	Active extract/fraction/compounds	Mechanism	Toxic dose	Material or mode	Study design	References
Hepatotoxicity						
Oxidative Stress	Bavachinin A	ROS accumulation and P38 and JNK proteins activation	IC50 = 11.97 μM	HepaRG cells	In vitro	[144]
	Isobavachalcone	ROS accumulation and Akt suppression	IC50 = 155.9 μM	HepG2 cells	In vitro	[145]
	Psoralen	Increased the expression and transcription levels of endoplasmic reticulum stress-related markers	80 mg/kg	Mice	In vivo	[147]
Cholestasis	Aqueous extracts of PCL	Induced cholestasis by PPAR α pathway	LD50 = 40.29 g/kg	Rats	In vivo	[149]
	Psoralen	Led to the disorder of bile acid transport and accumulation in hepatocytes by affecting bile acid transporter	60 mg/kg	Rats	In vivo	[150]
	8-Methoxypsoralen	Disrupted MDR3-mediated phospholipids efflux and bile acid homeostasis	200, 400 mg/kg; 100 μM	Rats; Human liver cell line L02	In vivo	[151]
	Bakuchiol	Promoted the expression of NTCP and resulted in the abnormal increase of bile acid concentration in hepatocytes	50 mg/kg	Mice	In vivo	[152]
Mitochondrial dysfunctions	Aqueous and ethanol extracts of PCL	Mitochondrial dysfunction-mediated apoptosis	5.14 g/kg	Mice	In vivo	[154]
	Bavachinin A	Damaged the structure and function of mitochondria	6.25, 12.5, 25 μM	HepaRG cells	In vitro	[155]
Metabolic disorders	Psoralen	Caused disturbances in amino acid metabolism, especially valine, leucine, and isoleucine biosynthesis in serum and liver	60 mg/kg	Rats	In vivo	[157]
	Isopsoralen	Induced disorders of phenylalanine, tyrosine, and glycine, serine, and threonine metabolism	3.5, 7.0, 14 mg/kg	Rats	In vivo	[158]
Abnormal liver regeneration	Psoralen	Upregulated cyclin E1 and p27, inhibited mTOR signaling and induced mitochondrial injury	LD50 = 1673 mg/kg; IC50 = 450 μM	Mice; L02 cells	In vivo and in vitro	[160]
Nephrotoxicity	70% ethanol extracts of PCL	Interfered with phospholipid metabolism, amino acid metabolism, purine metabolism and antioxidant system activity	0.54, 1.08, 1.62 g/kg	Rats	In vivo	[161]
	Furanocoumarins	Affected the renal organic ion transport system	20, 40 mg/kg	Mice	In vivo	[162]
	Bakuchiol	Damaged the cell membrane, triggered apoptosis and inhibited DNA synthesis in cells	40 μM	HK-2 cells	In vitro	[163]
Phototoxicity	8-Methoxyepsoralen	Metabolized slowly and accumulated in the serum and epidermis	3–5 mg/kg	Guinea pigs	In vivo	[167]
Developmental toxicity	Psoralen	Induced the oxidative stress, apoptosis, and energy metabolism Disorder	LC50 = 18.24 µM	Zebrafish Embryos/Larvae	In vivo	[168]
Reproductive toxicity	Ethanol extracts of PCL	Impaired testicular interstitial cell function and interfered the pituitary–testicular axis	10 g/kg	Rats	In vivo	[169]
	Psoralen	Decreased ovarian follicular function and reduced ovulation by enhancing estrogen metabolism levels	18, 90, 180 mg/kg	Rats	In vivo	[170]

## Hepatotoxicity

There have been an increasing number of reports of liver damage in recent years with the increase of PCL’s clinical use. According to studies, PCL-induced liver injury involves many mechanisms, such as oxidative damage, endoplasmic reticulum stress, cholestasis, mitochondrial dysfunction, metabolic disorders, and aberrant liver regeneration.

### Oxidative stress

Oxidative stress (OS), which is a condition of imbalance between the body’s oxidative and antioxidant effects, is a major factor in the emergence and progression of liver disorders [143]. In vitro research on the hepatotoxic components and mechanisms of PCL revealed that bavachinin A (IC50 = 11.97 μM) caused oxidative damage to HepaRG cells by damaging mitochondria, resulting in apoptosis and necrosis. The mechanism of toxicity may involve ROS accumulation and the activation of the P38 and JNK proteins [144]. Isobavachalcone (IC50 = 155.9 μM), according to another study, could cause cells to accumulate ROS, impair mitochondrial function, and decrease the phosphorylation of Akt, causing HepG2 cells to undergo apoptosis and death. As a result, reducing oxidative stress may be an effective clinical strategy for treating and preventing PCL-induced hepatotoxicity [145].

### Endoplasmic reticulum stress

In eukaryotic cells, the endoplasmic reticulum is a crucial organelle for protein synthesis, folding, and secretion. Endoplasmic reticulum function depends on the stability of its internal environment, but a variety of factors can cause an imbalance in endoplasmic reticulum homeostasis, which can lead to endoplasmic reticulum stress and then progress into inflammation, apoptosis, or steatosis through particular signal transduction pathways. Some studies have demonstrated that drug-induced endoplasmic reticulum stress can lead to hepatocyte apoptosis and liver injury [146]. In a study, mice’s liver coefficients significantly increased after receiving psoralen continuously for three days at a dose of 80 mg/kg, and the liver developed inflammatory cell foci and vacuolar degeneration. Additionally, the endoplasmic reticulum stress-related markers Grp78, PERK, eif2, ATF4, ire1, ATF6 and XBP1 expression and transcription levels significantly increased [147]. These findings suggested that psoralen could induce endoplasmic reticulum stress to cause liver damage.

### Cholestasis

Cholestasis refers to the disorder of secretion and excretion of bile acids, which is mainly manifested by the excessive accumulation of bile acids, cholesterol, bilirubin and other bile components in the liver and systemic circulation. Long-term persistent cholestasis can lead to liver fibrosis, cirrhosis, and even liver failure [148]. A study evaluated the hepatotoxic effects of aqueous extracts of PCL (LD50 = 40.29 g/kg), it showed that long-term administration could cause cholestatic hepatotoxicity, female rats were more sensitive to cholestatic hepatotoxicity, and PPARα may be the key pathway of cholestatic liver injury [149]. Psoralen (60 mg/kg) could cause liver damage by significantly increasing the liver weight ratio and liver coefficient, elevating serum levels of alanine aminotransferase (ALT), aspartate aminotransferase (AST), and total cholesterol, affecting bile acid synthesis and bile acid transporter, and disrupting bile acid transport and accumulation in hepatocytes [150]. An analysis of 8-MOP’s potential hepatotoxicity and effects on bile production revealed that it may cause cholestatic liver damage by interfering with multidrug resistance 3 P-glycoprotein (MDR3)-mediated phospholipids efflux and bile acid homeostasis [151]. Bakuchiol (50 mg/kg) could significantly promote the expression of the bile acid transporter sodium taurocholate cotransporting polypeptide (NTCP) in the liver of mice, which led to an aberrant rise in bile acid concentration in hepatocytes and damaged hepatocytes [152].

### Mitochondrial dysfunctions

Mitochondria are important organelles that maintain the physiological function of liver cells. A range of liver disorders are brought on by mitochondrial dysfunction, which results in cellular damages such ATP depletion, ROS accumulation, inflammatory response, Ca^2+^ compensation disorder, apoptosis, etc. [153]. In a study compared the hepatotoxicity of aqueous and ethanol extracts of PCL (5.14 g/kg) in mice, it was discovered that the ethanol extracts were substantially more hazardous to the liver than the water extracts. Bavachin, psoralidin, bavachinin, neobavaisoflavone and bakuchiol were the main hepatotoxic substances, and the mechanism of action may be related to oxidative stress and mitochondrial dysfunction-mediated apoptosis [154]. According to an in vitro study, bavachinin A could induce apoptosis or necrosis of HepaRG cells by damaging the structure and function of mitochondria, and at doses of 6.25 μM and 12.5 μM, the damage was mild and the cells were mainly apoptotic, while at doses of 25 μM, the damage was too severe and the cells were mainly necrotic [155].

### Metabolic disorders

The liver is an important place for the metabolism of sugars, proteins, fats, vitamins and hormones. Liver damage will lead to metabolic disorders, and metabolic disorders will also affect liver function [156]. A study found that psoralen (60 mg/kg) could disrupt the production of valine, leucine, and isoleucine in blood and the liver, causing harm to the liver [157]. Isopsoralen (3.5, 7.0, 14 mg/kg) could induce hepatotoxicity by disrupting the metabolism of amino acids such phenylalanine, tyrosine, and tryptophan as well as glycine, serine, and threonine. Further study revealed that the major routes involved in male rats were lipid and energy metabolism, while the main pathways affected in female rats were fatty acid metabolism [158]. Metabolomic studies suggested that PCL-induced liver damage was idiosyncratic, and its mechanism may have something to do with the regulation of the pathways for sphingolipid metabolism and tyrosine metabolism [159].

### Abnormal liver regeneration

The liver’s capacity for regeneration directly influences its capacity for self-healing and compensatory activities, and improper regeneration can result in a variety of liver lesions. A study reported that psoralen (In vivo: LD50 = 1673 mg/kg; In vitro: IC50 = 450 μM) could induce liver injury in mice. The decrease of liver regeneration and compensatory capacity caused by hepatocyte cycle arrest played an important role in the progression of hepatotoxicity, which may be related to the upregulation of cyclin E1 and p27, the suppression of mTOR signaling and mitochondrial damage [160].

## Nephrotoxicity

Numerous investigations have confirmed that PCL and its active components can lead to nephrotoxicity. The nephrotoxicity of 70% ethanol extracts of PCL (0.54, 1.08, and 1.62 g/kg) in rats was examined using UPLC-Q-TOF–MS serum metabolomics. It was discovered that the 70% ethanol extracts of PCL could interfere with phospholipid metabolism, amino acid metabolism, purine metabolism, and antioxidant system activity, leading to nephrotoxicity [161]. The furanocoumarins of PCL (20, 40 mg/kg) could interfere the metabolism, excretion and bioavailability of endogenous and exogenous compounds to damage kidney functions by affecting renal organic ion transport system [162]. Human proximal tubular epithelial (HK-2) cells exposed to bakuchiol (5, 10, 20, 30 and 40 μM for 4, 24, 48 and 72 h) demonstrated dose- and time-dependent toxic effects, with potential mechanisms of toxicity including direct cell membrane damage, triggering of apoptosis, inhibition of intracellular DNA synthesis, arrest of cell mitosis, and inhibition of cell proliferation [163]. Another study found that a single high-dose infusion of PCL (40 g/kg) caused kidney damage in rats, when PCL and Radix Glycyrrhizae were administered together, there was a tendency for the kidney damage to worsen [164].

## Phototoxicity

Phototoxic reaction is a non-immune reaction caused by the direct action of light energy on the skin, when a photosensitive chemical is present in the skin and is exposed to light of the proper wavelength and duration, it can result in a local or systemic toxic reaction [165]. PCL had strong photosensitive activity and could cause phototoxic contact dermatitis [166]. A study found that oral 5-methoxyepsoralen (5-MOP) caused phototoxic reactions at doses between 10 and 15 mg/kg while oral 8-MOP caused reactions at doses between 3 and 5 mg/kg. Therefore, switching to 5-MOP instead of 8-MOP could both fulfill the therapeutic goal and lower the risk of phototoxic anaphylaxis [167].

## Developmental and reproductive toxicity

Psoralen’s developmental toxicity was examined in a study on zebrafish embryos and larvae, it found that psoralen (LC50 = 18.24 µM) could increase the rate of deformity and decrease the hatchability and body length of zebrafish, and the toxic mechanism involved oxidative stress, apoptosis and abnormal energy metabolism [168]. Ethanol extracts of PCL (10 g/kg) could directly damage the function of testicular interstitial cells, interfere with the pituitary–testicular axis, rapidly reduce the level of androgen, and induce the injury of spermatogenic cells in seminiferous tubules [169]. Psoralen (18, 90, 180 mg/kg) could produce reproductive toxicity by increasing estrogen metabolism, reducing follicular function and ovulation [170].

In conclusion, the discovered toxicity of PCL mainly includes hepatotoxicity, nephrotoxicity, phototoxicity, developmental toxicity and reproductive toxicity, among which hepatotoxicity is most predominant, and psoralen, isopsoralen, bavachinin A and bakuchiol are the main toxic components. In recent years, many researchers have studied the methods of reducing the toxicity of PCL, and found that processing and compatibility are effective methods [171]. Hong et al. [172] study showed that the processing of PCL by Lei Gong method combined with salt could significantly reduce the hepatotoxicity of PCL, which may be related to the significant decrease in the contents of neobavaisoflavone, bavachin, isobavachalcone and bakuchiol, and slightly increase in the contents of psoralen and isopsoralen after processing. Another study found that compared with the raw PCL, salt PCL could significantly alleviate the side effects on the liver and kidney function of kidney-yang deficiency rats, which may be achieved by regulating the gene expression of aquaporin [173]. The study of compatibility and detoxification confirmed that Chinese magnolcavine fruit combined with PCL could alleviate the oxidative damage of hepatocytes and endoplasmic reticulum stress caused by PCL [174]. In addition, the compatibility of PCL with walnut kernel and semen myristicae could also reduce the hepatotoxicity and improve the liver injury caused by PCL, and the effect of compatibility of walnut kernel on reducing the hepatotoxicity of PCL was more obvious [175].

It is not difficult to find that psoralen, isopsoralen, bavachinin A and bakuchiol are not only the toxic component of PCL, but also its active component. Only by keeping the content of these ingredients within the range of effective dose can the safety and effectiveness of PCL be ensured. Therefore, in the future, the toxic substance basis, dose–effect-toxicity relationship and toxicological mechanism of PCL should be deeply studied, and scientific and effective methods of reducing toxicity should be sought to ensure the maximum efficacy while reducing the toxic reaction.

## Quality control

There are more than 120 species of genus psoralea, mainly distributed in southern Africa, North America, South America and Australia, a few in Asia and temperate Europe, and 1 species in China. According to the Chinese Pharmacopoeia, the leguminous plant *Psoralea corylifolia* L. is the only one officially permitted as a traditional source of herb. The shortage of original, genuine pharmaceutical materials caused by the rising demand for PCL has led to a growth in numerous counterfeit items, which has a significant negative impact on the quality of PCL. In addition, the origin, growth environment, harvest time and processing methods also lead to uneven quality of medicinal materials.

According to the literature records, the adulterated products of PCL mainly include semen abutili, datura seed, semen hyoscyami, and radish seed. Currently, the identification of PCL and its adulterated products is mostly based on the source, properties, physicochemical and microscopic. However, these conventional identification methods generally rely on subjective experience and have some limitations. In one study, PCL and its adulterants were successfully identified using DNA barcode technology to evaluate the ITS2 sequence. This method gets over the drawbacks of conventional techniques of identification, provides an objective identification basis directly from the genetic level, and is a good technological way to guarantee the caliber of PCL germplasm resources [176].

The Chinese Pharmacopoeia (2020 edition) controls the quality of PCL, stipulating that the total amount of psoralen and isopsoralen should not be less than 0.70% when PCL is calculated as dry product. However, the chemical component of PCL is diverse, only psoralen and isopsoralen are utilized as a quality control index, which is too limited and singular to accurately describe the integrity of its intricate system. To ensure the quality of PCL, it is vital to build straightforward, speedy, precise, and reliable quality control methods. In recent years, thin layer chromatography (TLC), performance liquid chromatography (HPLC), ultra-performance liquid chromatography (UPLC) and high-speed counter-current chromatography (HSCCC) have been widely conducted in qualitative analysis. Multi-components by single marker, multi-mode chemometrics and multi-component analysis methods are mainly used for quantitative analysis. A study used HSCCC fingerprint to control the quality of PCL, it was found that the chemical components of 20 batches of Chinese herbal medicines were similar, but their contents were quite different. At the same time, it was confirmed that the HSCCC approach had good reproducibility, precision, and stability, providing a solid foundation for the quality control of PCL and having the potential to be utilized to identify plant medicinal materials [177]. By combining the HPLC fingerprint and multi-component analysis, the quality of PCL was assessed. Based on the chromatographic analysis results of 28 batches of samples, 26 common peaks were found, 13 peaks were recognized, and the contents of these 13 compounds were calculated. In addition, principal component analysis was also used to examine and identify 28 batches of samples that were gathered from 6 different producing locations at various sampling times. The procedure is easy to use, rapid, and accurate, and it may be used to identify and monitor the quality of PCL from various harvesting periods and origins [178]. The UPLC fingerprint was established to evaluate the similarity of 29 batches of PCL from 10 producing areas. The results showed that the similarity of the 9 batches ranged from 0.827 to 0.989, 21 common peaks were identified and 12 of them were fingerprinted, and the content of 12 components was determined. The methodology verification found that this method is simple, accurate and stable, which can be used for qualitative and quantitative analysis of PCL and to provide a reference for the quality control of PCL and associated preparations [75]. A more effective way to assess the comprehensive quality of PCL from different regions is made possible by the recently established quantitative analysis of multi-components by single marker for simultaneous determination of 16 compounds in PCL [179].

The most important factor in ensuring the security and effectiveness of clinical use is the quality of Chinese medicinal herbs, which needs to be strictly controlled. Chinese medicinal herbs have the characteristics of multiple components, multiple targets and multiple pathways, there are obvious limitations in reflecting the quality of Chinese medicines with only one or two compounds. Therefore, the scope of compositional studies should be expanded in the future to better characterize the quality integrity of PCL complex systems. Additionally, the methods used for processing, extracting, determining, and storing PCL are significant elements affecting its quality standard. Therefore, in order to accurately reflect the quality of medicinal materials, these aspects should be completely taken into account while establishing the PCL quality standard in the future.

## Pharmacokinetics

The most crucial step in assessing whether new drugs are effective is pharmacokinetics. Research on the dynamic changes of absorption, distribution, metabolism and excretion of PCL and its chemical components in vivo can serve as a guide for medication development and clinical application. A study compared the pharmacokinetic differences of isopsoralen in female and male rats, it found that female rats had greater AUC0-t, AUC0-∞, Cmax values, and lower CLZ/F values than male rats, suggesting that isopsoralen may accumulate more readily in female rats, and female rats were more susceptible to the toxicity of isopsoralen [180]. Another study used UHPC-MS/MS to analyze the pharmacokinetic behavior of 17 components in the serum of rats after oral administration of aqueous and ethanol extracts (1.0, 2.0 g/kg) of PCL. Nine bioactive components were identified in 17 target analytes within 36 h of treatment. Further research revealed that the Cmax and AUC of ethanol extracts of PCL (EPC) administration were much lower than aqueous extracts of PCL (APC) and that the EPC content was at least ten times higher than that of the APC. The relative diversity and intricate interplay of bioavailable EPC constituents in the absorption and elimination phases may result from this phenomenon [181]. An LC–MS/MS method was established to study the pharmacokinetics of 11 analytes in rat plasma after oral administration of PCL extracts, the results showed that all the compounds were absorbed into the blood and rapidly distributed to the brain. Moreover, the plasma concentrations (AUC 0 → ∞, plasma ≈ 53,884–65,578 ng h/mL) and central nervous system penetration (AUC 0 → ∞, brain nuclei ≈ 44,659–65,823 ng h/g) of the coumarins were much higher than those of the prenylflavonoids (AUC 0 → ∞, plasma ≈ 69–324 ng h/mL; AUC 0 → ∞, brain nuclei ≈ 119–3662 ng h/g), but the total brain-to-plasma ratio of prenylflavonoids was higher than those of coumarins, suggesting that prenylflavonoids were more likely to penetrate the blood–brain barrier and accumulate in the brain [182]. The pharmacokinetics, tissue distribution and excretion of psoralen and isopsoralen after oral (9.12 mg/kg) and intravenous (2.00 mg/kg) administration were investigated by LC–MS/MS, it was found that psoralen and isopsoralen had a high oral bioavailability, and that their elimination half-lives after oral administration were 4.13 h and 5.56 h, the relative oral bioavailability were 61.45% and 70.35%. After intravenous administration, psoralen and isopsoralen could be rapidly and widely distributed to most tissues, but they couldn’t effectively pass through the blood–brain barrier and were slowly cleared and excreted, mainly through urine [183]. Another study discovered that salt-processed could significantly promote the absorption of psoralen and isopsoralen, and improve the bioavailability of the compounds [184]. LC–MS/MS in combination with MRM mode was developed to determine the pharmacokinetics of bakuchicin in mouse plasma, the study’s findings revealed that the retention time of bakuchiol was 4.5 min, the concentration range of 20–1000 ng/mL showed a good linear relationship (r^2^ = 0.996), the lower limit of quantification in mouse plasma was 20 ng/mL, and the bioavailability of psoralen when administered orally at 5 mg/kg was 58.3% [185].

## Conclusion and future prospects

This paper reviews the research progress in the botany, traditional uses, phytochemistry, pharmacology, toxicology, quality control and pharmacokinetics. At present, the components isolated from PCL mainly include coumarins, flavonoids, monoterpene phenols, benzofurans, lipids, fatty acids, glycosides and volatile oils. PCL has anti-inflammatory, antibacterial, antioxidant, anticancer, photosensitive activity, estrogen-like effect and other pharmacological activities. At the same time, it also shows high medicinal value in regulating the functions of cardiovascular system, nervous system, immune system and motor system. Safety studies have shown that long-term or excessive use of PCL and its active ingredients can cause hepatotoxicity, nephrotoxicity, phototoxicity, developmental toxicity and reproductive toxicity. In recent years, some achievements have been made in the study of chemical composition, pharmacological activity, toxicity and quality control of PCL. However, more investigations are required to have a thorough and in-depth understanding of PCL. Therefore, we put forward several directions for further research in the future.

First, the chemical compositions of PCL are complex, but the current studies on these compounds are mainly focused on coumarins, flavonoids and monoterpene phenols, while there are relatively few studies on benzofurans, lipids and fatty acids. Chemical components are the basis for the pharmacological effects of drugs, and other components also need to be extensively developed and researched. In the future, we can try new separation and determination methods to explore and analyze other components in PCL, so as to enrich the material basis of PCL and avoid the underutilization of effective ingredients.

Second, there are more and more reports about the toxicity of PCL, such as hepatotoxicity, nephrotoxicity, phototoxicity, developmental toxicity and reproductive toxicity. Among them, liver injury is the most important toxic reaction. At present, it is considered that psoralen, isopsoralen, bakuchiol and bavachinin A are the main toxic components, but the minimum effective dose, minimum toxic dose and minimum lethal dose of these components are still uncertain. The material basis, dose–effect-toxicity relationship and toxicological mechanism of PCL need to be studied comprehensively and deeply. In addition, it is also extremely important to seek scientific and effective methods to reduce toxic reactions and maximize pharmacological effects.

Third, in terms of quality control, a single method for determining the content of a specific kind of component or even just a single component may not accurately reflect the quality of PCL. Therefore, to accomplish more comprehensive quality control, future studies can focus more on the simultaneous assessment of many components. The methods used for extracting, processing, determining, and storing are also significant elements affecting the PCL quality. Therefore, it is recommended that these aspects be fully taken into account when the PCL quality standards are developed in the future.

Fourth, the relationship between the traditional uses of PCL and its modern pharmacological activity has not been fully investigated. The traditional uses of PCL are reflected in clinical practice, while pharmacological studies are mostly based on animal and cell experiments, clinical application and experimental research cannot be well combined. Future research should focus on developing disease-syndrome combination models and standards for evaluation models so that the experiment can effectively direct clinical drug use.

Fifth, oral and injectable delivery of PCL result in distinct pharmacokinetic behaviors. Therefore, it is important to increase the comparative analysis of them. Chemical components are the material basis for the efficacy of drugs. Clarification of the dynamic changes in absorption, distribution, metabolism, and excretion of active components in the body is necessary for the creation of new drugs and the enhancement of bioavailability.

Sixth, PCL is typically combined with other medications in clinical practice rather than being used alone to treat diseases. However, the mechanism of PCL alone or in combination with other medications, as well as the changes of chemical composition and efficacy before and after combined application, are not well studied and need to be further explored. This is crucial to elucidating the compatibility of PCL.

In conclusion, this paper comprehensively reviewed the botany, traditional uses, phytochemistry, pharmacology, toxicology, quality control and pharmacokinetics of PCL, and prospected the future research directions of PCL, in order to provide valuable references for the development and rational application of PCL in the future.

## Data Availability

The available information on PCL was collected from scientific databases, including PubMed, Google Scholar, Baidu Scholar, Web of Science, SciFinder, Springer, ScienceDirect, Chinese Biomedical Database (CBM) and CNKI, and classic books on Chinese herbal medicines were also searched.

## Abbreviations

AIM2: Absent in melanoma 2
AKT: Protein kinase B
AD: Atopic dermatitis
APP: Amyloid precursor protein
AP-1: Activator protein 1
Axin2: Axis inhibition protein 2
ATF6: Recombinant activating transcription factor 6
ALT: Alanine aminotransferase
AST: Aspartate aminotransferase
ATP: Adenosine triphosphate
APC: Aqueous extracts of *Psoralea corylifolia* L.
AChE: Acetylcholinesterase
BACE1: β-Secretase
Bcl-2: B-cell lymphoma-2
Bax: Bcl-2X-associated protein
BDNF: Brain-derived neurotrophic factor
BHK21: Baby hamster kidney fibroblast cell line
CBM: Chinese Biomedical Literature Database
COX-2: Cyclooxygenase-2
cGMP: Cyclic guanosine monophosphate
CD107a: Lysosome-associated membrane protein-1
CTR: Copper transport protein
CHOP: CCAAT/enhancer-binding protein homologous protein
DENV-1: Dengue virus type 1
ED50: 50% Effective dose
EX527: Selisistat
EC90: Concentration for 90% of maximal effect
ERK: Extracellular regulated protein kinases
ERα: Estrogen receptor α
ERβ: Estrogen receptor β
ER: Estrogen receptor
eNOS: Endothelial nitric oxide synthase
eIF2α: Eukaryotic initiation factor 2
EPC: Ethanol extracts of *Psoralea corylifolia* L.
FOXO1: Recombinant forkhead box protein O1
FSHR: Follicle stimulating hormone receptor
GSH-Px: Glutathione peroxidase
GSK-3β: Glycogen synthase kinase-3β
GZMB: Granzyme B
Grp78: Glucose regulated protein 78
HBV: Hepatitis B virus
HaCaT: Human immortal keratinocyte line
HUVECs: Human umbilical vein endothelial cells
HPA: Hypothalamic–pituitary–adrenal
HO-1: Heme oxygenase 1
iNOS: Inducible nitric oxide synthase
IL: Interleukin
IGF-1R: Insulin-like growth factor 1
IRAK4: Recombinant interleukin 1 receptor associated kinase 4
Itgb1: Integrin B1
ire1α: Inositol-requiring enzyme 1α
iBMDMs: Bone marrow-derived macrophages
JNK: C-jun N-terminal kinase
LPS: Lipopolysaccharide
LHR: Luteinizing hormone receptor
MIF: Macrophage migration inhibitory factor
MHV-68: Murine gamma herpesvirus 68
MDA: Malondialdehyde
MCF-7: Human breast cancer cell line
MMPS: Matrix metalloproteinases
8-MOP: 8-Methoxy-psoralen
5-MOP: 5-Methoxy-psoralen
MAO: Monoamine oxidase
MAPK: Mitogen-activated protein kinase
M-CSF: Macrophage colony-stimulating factor
MITF: Membership implementation task force
MDR3: Multidrug resistance 3 P-glycoprotein
NLRP3: Nod-like receptor protein 3
NLRC4: NOD-like receptor C4
NF-κB: Nuclear factor kappa-B
Nrf2: Nuclear factor erythroid 2-related factor 2
NGF: Nerve growth factor
NBUVB: Narrow-band ultraviolet B
NO: Nitric oxide
NTCP: Sodium taurocholate cotransporting polypeptide
NRVMs: Neonatal rat ventricular myocytes
OCT-4: Octamer-binding protein 4
PCL: *Psoralea corylifolia* L.
PUVA: Psoralen combined with UVA
PI3K: Phosphatidylinositide 3-kinases
PGC-1α: Peroxisome proliferator-activated receptor γ coactivator-1α
PCp-I: Polysaccharide
PPAR-γ: Peroxisome proliferators activate receptors-γ
PERK: Protein kinase RNA-like endoplasmic reticulum kinase
PF: Perforin
ROS: Reactive oxygen species
RSK2: Ribosomal protein S6 Kinase α3
RANKL: Receptor activator of nuclear factor-κB ligand
RANK: Receptor activator of nuclear factor-κB
SIRT1: Silent information regulator 1
SARS-CoV: Severe acute respiratory syndrome coronavirus
SOD: Superoxide dismutase
Smad3: Recombinant SMAD family member 3
Stra8: Stimulated by retinoic acid gene 8
SCP3: Synaptonemal complex protein 3
SOCS-3: Recombinant suppressors of cytokine signaling 3
α-SMA: α- Smooth muscle actin
Smurf2: Smad ubiquitination regulatory factor 2
TNF-α: Tumor necrosis factor α
TCMs: Traditional Chinese medicines
TLR4: Toll-like receptor 4
TRPC3: Transient receptor potential channel
TGF-β: Transforming growth factor-β
TRAF6: Tumor necrosis factor receptor-associated factor 6
TRAP: Triiodothyronine receptor auxiliary protein
TYR: Tyrosinase
TRP-1: Tyrosinase related protein-1
TRP-2: Tyrosinase related protein-2
UVC: Ultra-Violet C
UVA: Ultra-Violet A
UVB: Ultra-Violet B
uPA: Urokinase type fibrinolytic enzyme former activators
XBP1: Spliced form of X-box binding protein1
